# At the beginnings of the funerary Megalithism in Iberia at Campo de Hockey necropolis

**DOI:** 10.1038/s41598-022-13014-6

**Published:** 2022-06-08

**Authors:** Eduardo Vijande-Vila, Marta Díaz-Zorita Bonilla, Berta Morell-Rovira, Íñigo Olalde, Lydia P. Sánchez-Barba Muñoz, Salvador Domínguez-Bella, Steven D. Emslie, Serafín Becerra-Martín, Ángel Rubio-Salvador, Diego Salvador Fernández-Sánchez, Juan Jesús Cantillo-Duarte, Inmaculada Alemán-Aguilera, Adolfo Moreno-Márquez, Eduardo Molina-Piernas, José Luis Ramírez-Amador, María Leticia Gómez-Sánchez, Miguel C. Botella-López, Joaquín Rodríguez-Vidal, José Ramos-Muñoz

**Affiliations:** 1 Área de Prehistoria, Universidad de Cádiz, Avda. Dr. Gómez Ulla, 1, 11003 Cádiz, Spain; 2 Institut für Ur- und Frühgeschichte und Archäologie des Mittelalters, Burgsteige 11, 72070 Tübingen, Germany; 3 Departamento de Historia, Arte y Geografía, Facultad de Historia, Pabellón 1, Universidad de Vigo, 1º Planta. Campus As Lagoas s/n, 32004 Ourense, Spain; 4 Max Planck Institute for the Science of Human History, Kahlaische Strasse 10, 07745 Jena, Germany; 5 BIOMICs Research Group, University of the Basque Country UPV/EHU, Vitoria-Gasteiz, Spain; 6 Ikerbasque—Basque Foundation of Science, Bilbao, Spain; 7 Departamento de Medicina Legal, Toxicología y Antropología Física, Universidad de Granada, Avda. de la Investigación 11, 18016 Granada, Spain; 8 Unit of Geoarchaeology and Archaeometry Applied to the Historic-Artistic and Monumental Heritage (UGEA-PHAM), Department of Earth Science, Faculty of Sciences, University of Cádiz, Av. República Árabe Saharaui s/n, Puerto Real, 11510 Cádiz, Spain; 9 Department of Biology and Marine Biology, University of North Carolina, 601 S. College Rd., Wilmington, NC 28403 USA; 10 Universidad de Jaén, Departamento de Antropología, Geografía e Historia, Campus de Las Lagunillas s/n, 23071 Jaén, Spain; 11 Department of Geography, History and Humanities, University of Almería, Almería, Spain; 12 Departamento de Ciencias de la Tierra., Universidad de Huelva, Avda. Tres de Marzo, s/n, 21071 Huelva, Spain

**Keywords:** Anthropology, Archaeology, Population genetics

## Abstract

The excavations undertaken at the Campo de Hockey site in 2008 led to the identification of a major Neolithic necropolis in the former Island of San Fernando (Bay of Cádiz). This work presents the results of the latest studies, which indicate that the site stands as one of the oldest megalithic necropolises in the Iberian Peninsula. The main aim of this work is to present with precision the chronology of this necropolis through a Bayesian statistical model that confirms that the necropolis was in use from c. 4300 to 3800 cal BC. The presence of prestige grave goods in the earliest and most monumental graves suggest that the Megalithism phenomenon emerged in relation to maritime routes linked to the distribution of exotic products. We also aim to examine funerary practices in these early megalithic communities, and especially their way of life and the social reproduction system. As such, in addition to the chronological information and the Bayesian statistics, we provide the results of a comprehensive interdisciplinary study, including anthropological, archaeometric and genetic data.

## Introduction

Much has been written about the origins of European megalithism. Between the late 19th and the mid-twentieth centuries, orientalist diffusionist theories put forward by archaeologists such as Montelius^1^ and Childe^2^ constituted the dominating paradigm. Some authors, however, located the origin of megalithism in western Europe, for instance Bosch Gimpera, who argued that Portuguese single dolmen graves, built by Mesolithic shepherds, were the earliest megaliths^3,4^, or Georg and Vera Leisner who accepted the exceptional nature and antiquity of square-shaped and trapezoidal Portuguese megalithic chambers^5^. Daniel, however, linked this phenomenon with Atlantic Mesolithic communities, who were behind the construction of large necropolis of stone mound-covered cist-graves, such as Téviec, in Morbihan^6^. However, the oriental paradigm remained dominating until the 1960s^7^, when radiocarbon dating confirmed the greater antiquity of western megaliths^8–11^. Despite this, dating the beginning of this phenomenon with any precision remains tricky, although recent studies^12^ suggest an origin in the first half of the 5th millennium BC in French Brittany, whence it rapidly spread (by sea) to the Mediterranean and the Atlantic coast of the Iberian Peninsula.

In the Iberian Peninsula, funerary megaliths dated to between the mid-4th and the early 2nd millennia are well known. However, the origins of the phenomenon could be probably dated back to the second half of the 5th millennium BC, and it is still poorly defined. Few proto-megalithic sites are known, and in those that exist human remains are either lacking or badly preserved, or the archaeological contexts have been ruined by looting and other activities. In addition, few human remains have been ^14^C-dated (AMS), and virtually none of these dates have been subject to Bayesian statistical analysis.

In Andalusia (southern Spain), the 5th millennium BC is poorly known. Funerary contexts are scarce, and most of them have been found in caves^13,14^. The earliest open air necropolises are dated to the late 5th and early 4th millennia BC^14,15^, along with the emergence of the first permanent settlements, as illustrated by the Campo de Hockey site^16,17^. This marks the beginning of the funerary Megalithism in the Iberian Peninsula, specifically Phase I (proto-megalithism), characterised by small chamber tombs or burial mounds, occupied by an individual or a small group, and scarce grave goods. The phenomenon implies the adoption of new burial practices involving the use of stone architecture. In contrast with the large monumental constructions that characterise the central phase of Megalithism, the proto-megalithic phase is characterised by the erection of (mostly) one-use small chamber structures without access corridors.

The Campo de Hockey necropolis displays a type of funerary expression that has no parallels in the Neolithic of the southern Iberian Peninsula; it is a large necropolis, with a large number of burials, most of which are individual, and a wide variety of constructive typologies. For these reasons, the main objective of this work is to increase our understanding of early funerary Megalithism in the Iberian Peninsula, with special emphasis on: (1) establishing the chronology of the necropolis and of the most complex—proto-megalithic—burials; (2) identifying patterns in terms of orientation, and the possible segregation of space according to sex, age and tomb typology, and; (3) establishing the chronology of exotic and prestige grave goods, which were mostly found in the most monumental burials, with the aim of determining distribution and circulation routes. To achieve this, radiocarbon dates were obtained from human bone (short life samples) applying Bayesian statistical analyses. Additionally, interdisciplinary architectural, anthropological, genetic, archaeometric, and bio-archaeological data were systematically collected to better understand funerary ritual and practices, as well as lifestyle, production and social reproduction within the community.

## Archaeological background: the Campo de Hockey settlement

**Figure 1:**
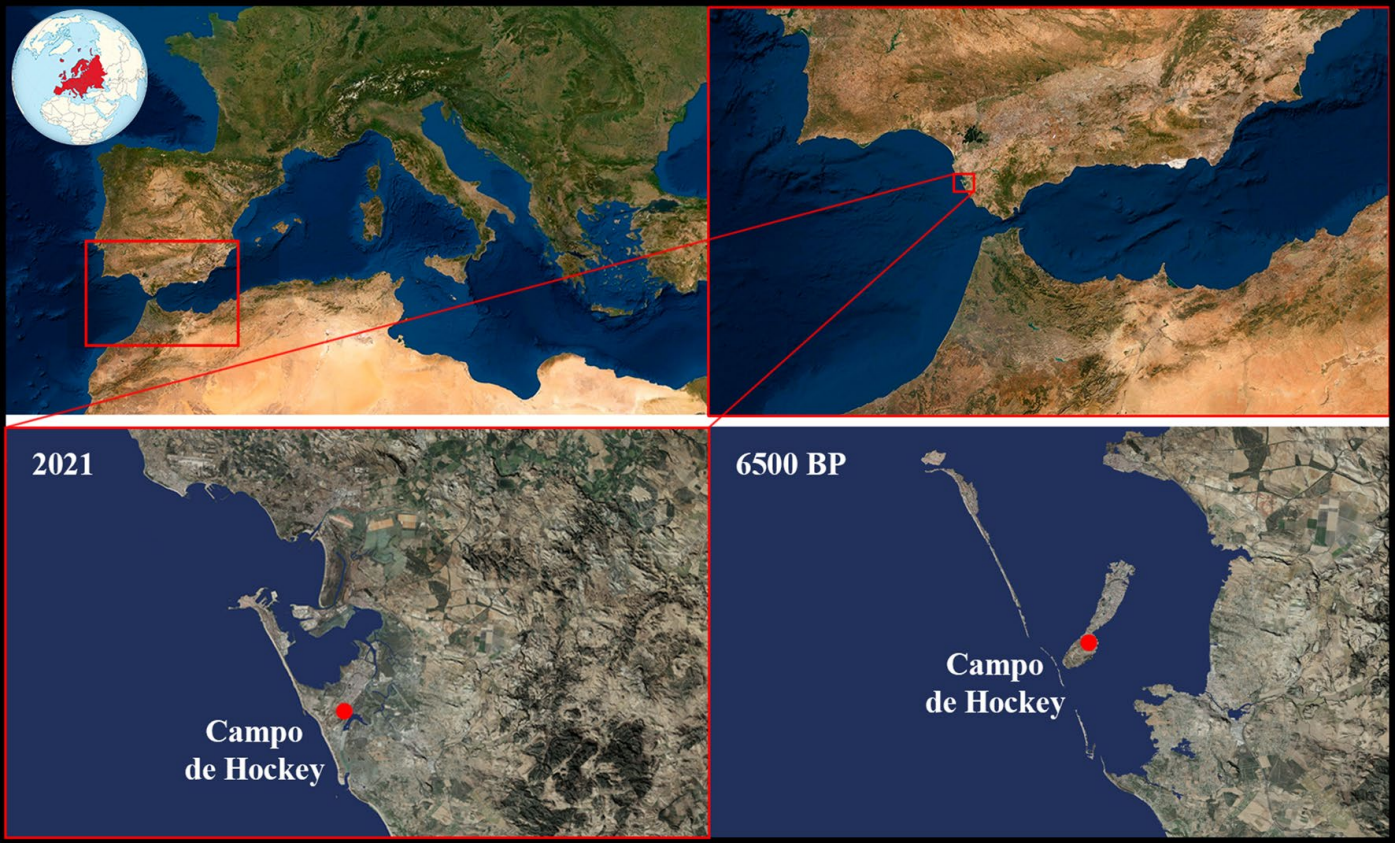
Geographical setting of the Campo de Hockey site, and paleo-geographical reconstruction of the Bay of Cádiz 6500 BP^15^. Satellite images were obtained using SAS.Planet version 190707 (freeware with GNU General Public Licence) (http://www.sasgis.org/). Photo setting was design with the application of Adobe Photoshop version CC 2021 (22.3.1) (http://www.adobe.com).

The Neolithic settlement of Campo de Hockey is located south of the municipality of San Fernando, which was an island in the Bay of Cádiz during the Neolithic period (Fig. 1)^18,19^. The convergence of the Mediterranean and the Atlantic in the Strait of Gibraltar makes for a very rich marine environment, which local Neolithic communities exploited thoroughly^20^. The archaeological excavation, undertaken between August 2007 and July 2008, revealed domestic structures (hut floors and hearths), storage features (well-silos) and a large necropolis in the easternmost sector of the site, which is the focus of this work^16^.

Campo de Hockey is one of the earliest permanent open air settlements in the southern Iberian Peninsula. It is located on a slope 12–18 m.a.s.l., barely 150 m from the ancient coastline^16,17^ (Fig. 1). Palynological and anthracological studies suggest an open Mediterranean landscape populated by trees and bushes for the earliest period and a herbaceous environment for the later period, the result of deforestation caused by approximately 300 years of intense human exploitation^21^. The archaeological record indicates that the community’s economy was based on agriculture, livestock and intensive use of marine resources, as reflected by the rich malacological and ichthyological assemblage^20^.

The excavation of the necropolis resulted in the identification of 53 tombs and 63 buried individuals—45 individual graves, seven double graves and one quadruple grave. The unarticulated remains of another ten individuals were found scattered in the fills, the result of post-depositional processes and ancient soil-shifting operations. The funerary ritual followed was inhumation (47 bodies were found anatomically articulated), including 32 bodies lying on the right side and fifteen on their left side.

## Methods

### Tomb typology

This study introduces and updates the tomb typologies previously published^16^. Typologically, the tombs can be divided into eight types and four subtypes, according to structural complexity, morphology and construction materials (pits and stone). In this regard, it should be noted that 64.2% of the tombs (34 of 53) belong to Type I (no stone was used in their construction), while those built using large stones (proto-megalithic; Types V, VI, VII and VIII), barely made up to 7.6% of the total (Table 1).

**Table 1:** Distribution of the different types of tombs by trench in the Campo de Hockey necropolis.

Type	Trench 7	Trench 9	Trench 12	Trench 13A	Trench 13B	Trench 16	Trench 14	Trench 15	Trench 17	Total
I	–	–	2	3	3	4	12	6	4	34
IIA	–	1	–	1	–	–	–	3	–	5
IIB	–	–	–	1	–	–	–	1	–	2
IIC	–	–	–	–	1	–	1	1	1	4
III	–	–	1	–	–	–	–	–	–	1
IIIA	–	–	1	–	–	–	–	–	–	1
IV	–	–	–	1	1	–	–	–	–	2
V	1	–	–	–	–	–	–	–	–	1
VI	–	–	–	1	–	–	–	–	–	1
VII	–	–	–	–	–	–	–	1	–	1
VIII	–	–	–	–	–	–	1	–	–	1
Total	1	1	4	7	5	4	14	12	5	53

### Radiocarbon dates and Bayesian analysis

A total of nineteen radiocarbon dates were obtained, 14 of which are presented in this paper (Table 2). Fifteen dates correspond to human bone samples from fifteen different tombs (20.83% of the total number of documented individuals and 28.3% of the graves) and four to marine shells (*Mondonta lineata*, *Bolinus brandaris* and *Phorcus lineatus*) from two different burials, a hearth (located in the south-eastern part of the site, trench 17) and a single pit documented in the western sector, outside of the cemetery (trench 2, UE 205). All the samples came from reliable contexts conformed mainly by primary inhumations with articulated skeletons in a good state of preservation and well-characterized structures. The radiocarbon sampling strategy has taken into consideration the minimum number of individuals, the estimation of the sex and the absence/presence of grave goods in relation to the spatial distribution of the features.

**Table 2:** Radiocarbon dates from the Campo de Hockey necropolis.

Lab.Code	14C date	es.Dv	Sample	Structure and individual	Type of structure	Grave good	% Marine	Cal.BC (2σ)	References
ETH-107763	5258	27	Human bone	E10T13A Ind. 9	IV	No	4	4225–3970	Unpublished
CNA-4579.1.1	5140	35	Human bone	E16T15 Ind.11	IIb	No	22	3960–3715	Unpublished
ETH-107764	5186	24	Human bone	E6T12 Ind.21	I	No	2	4040–3945	Unpublished
ETH-88972	5364	24	Human bone	E11T14 Ind.16	VIII	Yes	40	4060–3950	^83^
ETH-107765	5350	24	Human bone	E14T15 Ind.36	I	No	45	4045–3920	Unpublished
ETH-107766	5178	25	Human bone	E6T13B Ind.28	IIc	No	7	4040–3810	Unpublished
ETH-107767	5093	24	Human bone	E5T15 Ind.12	IIc	No	22	3935–3700	Unpublished
CNA-4581.1.1	5250	35	Human bone	E8T15 Ind.13	I	No	33	3990–3800	Unpublished
ETH-107768	5262	25	Human bone	E3T17 Ind.43	I	No	40	3970–3800	Unpublished
ETH-107769	5270	26	Human bone	E6T15 Ind.8-52	IIa	No	18	4050–3950	Unpublished
ETH-107770	5120	26	Human bone	E7T13B Ind.8-30	IV	No	18	3950–3770	Unpublished
ETH-107771	5245	24	Human bone	E4T16 Ind.8-55	I	Yes	40	3960–3800	Unpublished
BETA-564528	5410	30	Human bone	E3T15 Ind.41	VII	Yes	4	4335–4070	Unpublished
BETA-569324	5250	30	Human bone	E4T13A Ind.10	VI	Yes	21	4045–3820	Unpublished
CNA-360	5020	50	Human bone	E10T15 Ind.75/76	I	No	3	3925–3635	^17^
CNA-833	5665	50	Shell. *Murex Brandaris*	E7T7	V	No	100	^4060–3665^	^17^
CNA-835	5485	30	Shell. *Monodonta lineata*	Trench 2-UE 205	Domestic pit	–	100	3875–3515	^17^
CNA-664	5650	40	Shell. *Phorcus lineatus*	E11T14	VIII	–	100	4036–3669	^17^
CNA- Non det	5805	48	Shell *Monodonta lineata*	T17/UE1712	Hearth	–	100	4235–3830	Unpublished

The type of funerary structure, the presence/absence of grave goods and the calculated percentage of marine consumption are indicated.

Prior to radiocarbon analyses, elemental analyses were completed (%C and %N) as an indicator of collagen preservation. Samples of %N < 0.40 were discarded from the analysis. Therefore, results indicated carbon and nitrogen elemental composition in whole %C from 4.11 to 9.78 and for %N samples bone ranged from 0.46 to 1.23.

Collagen yields for carbon and nitrogen ranged from 17.71% to 32.68% and 6.14% to 11.29%, respectively (Table 2). The atomic C/N ratio ranged from 3.3 to 3.6 with a mean of 3.4 and all samples presented acceptable C/N ratio^22^. To investigate the potential reservoir effect, stable isotope analyses of *δ*^13^C and *δ*^15^N were also undertaken. This was corrected through the mixed terrestrial (*IntCal20*^23^) and marine (*Marine20*^24^) calibration curve. Seashell determinations were calibrated with the *Marine20* calibration curve, assuming a 100% reservoir effect incidence. The possible regional differences of the curve were mitigated through the ∆R parameter. An average of 32.63 ± 33.27 was estimated, based on the recorded values (within a radius of 100 km of the site) available in the free access IsoMemoApp Database^25^. The % of marine consumption was estimated using food source data of marine and terrestrial fauna samples from Neolithic to contemporary nearby sites^26^. All the radiocarbon dates were calibrated with *OxCal v.4.4* software^27^.

All radiocarbon dates were analysed from different Bayesian Models^27^ and the Chi-Square Test^28^ through *OxCal v4.4.* software, not only to determine the chronology of the site but also to explore the temporal distribution of certain elements and patterns. Specifically, the aims of the chronological analysis were: (1) to determine the start date, duration and end date of the use of Campo de Hockey necropolis; (2) to explore to what an extent the variability in the presence and absence of grave goods and non-local materials (amber, green stone beads and sillimanite) corresponded to chronological issues or other factors, and; (3) to test if there is a chronological factor in the spatial distribution of the graves within site and the different types of funerary structures.

### Bioarchaeology

A total of 72 individuals were examined to estimate sex and age. The sex of adult individuals was determined through the examination of their skull and pelvic bones^29,30^. The sex of many individuals could not be determined due to their poor state of preservation. In these cases, post-cranial elements were measured to estimate the sex through discriminating functions^31^, or based on the size and robustness of long bones, as sexual dimorphism was very pronounced in this community. On the other hand, age was determined by the degree of synostosis in cranial sutures^32^, changes in the pubic symphysis^33^ and pelvic auricular surfaces^34^.

Although the sex of subadult individuals could not be estimated due to the lack of the bone structures necessary for their discrimination (mandible and ilium)^35,36^, age could be estimated based on bone maturity criteria and dental growth^37,38^. The individuals were divided into the following age groups: neonates (0–6 months); infants I (6 months–7 years); infants II (7–14 years); juveniles (14–21 years); adults (21–40 years); matures (41–60 years); and seniles (≥ 61 years).

### DNA

Genome-wide data from six individuals from Campo de Hockey were generated as part of this project (Olalde et al*.*^39^) and can be downloaded from the European Nucleotide Archive database (accession PRJEB30874). Three individuals yielded a better DNA preservation and between ~ 260,000–660,000 autosomal genetic markers (SNPs) could be recovered, while the remaining three yielded less than 100,000 markers. Complete mitochondrial genomes were also generated for the six individuals.

To determine the genetic sex of the Campo de Hockey individuals, we used the method described in Skoglund et al*.*^40^, which computes the ratio between the DNA sequences mapped to the Y-chromosome and the sum of DNA sequences mapped to X- and Y-chromosomes. Given that females lack the Y-chromosome, their expected ratio is close to 0, while males show ratios larger than 0.30 for this type of data.

To study kinship relationships through all genealogical lines, the autosomal markers were analysed. First, the mismatch rate at autosomal markers for each pair of individuals was computed, by randomly sampling one reading at each SNP for each individual^39,41,42^. Then, we normalized these values by the mismatch rate expected for two unrelated Iberian Neolithic individuals and estimated the coefficient of relatedness (equivalent to the portion of the genome being shared) using the method used by Kennett et al*.*^41^ and Olalde et al*.*^39^.

To explore the genetic affinities of the individuals from Campo de Hockey, a Principal Component Analysis (PCA) was performed on 591,642 autosomal SNPs using the ‘smartpca’ program in EIGENSOFT^43^. Then, ancient individuals from Campo de Hockey and from other relevant archaeological sites were projected onto the components computed on 989 present-day West Eurasian individuals (not shown in the PCA figure) genotyped on the Human Origins Array^44–46^, with options lsqproject: YES and shrinkmode: YES. We also used the f-statistics framework implemented in ADMIXTOOLS^46^ to test for differential genetic affinity to Mesolithic hunter-gatherers, and the qpAdm program^47^ (https://github.com/DReichLab) to estimate ancestry proportions.

We explore the presence of endogamy among the Campo de Hockey individuals through the detection of Runs of Homozygosity (ROH). ROH segments are stretches of an individual’s genome that show identical alleles on both homologous chromosomes, whereby both copies of the genome have been inherited from the same recent common ancestor. Studying the length and number of ROHs can give information about the degree of relatedness between the parents of a given individual. To identify ROH segments, we ran hapROH^48^ on the two higher-coverage individuals (I7550 and I8134). Additionally, we applied the HIrisPlex-S system^49^ to predict skin, hair and eye colour. For each individual, we retrieved the alleles present at the 41 SNPs included in HIrisPlex-S, and upload the genotypes to the https://hirisplex.erasmusmc.nl/ website for prediction.

### Grave goods

Exotic grave goods were analysed by X-ray diffraction, X-ray fluorescence spectrometry, and Fourier transform infrared spectroscopy. X-ray diffraction is one of the most suitable methods for the detection of the mineral phases. In this case, due to their characteristics, the flattest surfaces in the archaeological artefacts under analysis (polished adzes and axes, necklace and pendant beads) were directly exposed to the non-destructive X-rays, allowing the minerals present in the object to be detected without reducing the sample to powder. The analyses were conducted with a Bruker D-8 Advance ECO diffractometer (hosted by the Servicios Centrales de Investigación Científica y Tecnológica (SC-ICYT), Universidad de Cádiz), equipped with a high-speed measurement Link-Eye detector using the following specifications: Cu K alfa radiation filtered by Ni, graphite monocromator and fixed slots, 2theta 5° to 60° scanning angle; diffractograms were interpreted with the software EVA (Bruker-AXS).

X-ray fluorescence is a valuable method for the semi-quantitative and quantitative analysis of solid mineral and metal samples. It can be used on both powdered and solid samples, and it is non-destructive. The analyses were undertaken with a Bruker AXS M-4 Tornado sequential dispersive wave X-ray spectrometer (hosted by Servicios Centrales de Investigación Científica y Tecnológica (SC-ICYT), University of Cádiz), with a Rh tube operating at 4000 W.

Fourier transform infrared spectroscopy is a precise technique for the determination of both organic and inorganic compounds. The samples studied here were previously analysed by Vijande et al*.*^17^. In this instance, alteration areas were avoided to prevent noise. A small amount of matter from the inside of beads was taken to prepare KBr pellets, which were analysed with a Bruker Alpha II device (hosted by Unit of Geoarchaeology and Archaeometry Applied to the Historic-Artistic and Monumental Heritage (UGEA-PHAM), University of Cádiz), using a spectral range between 4000–400 cm^−1^, a resolution of 2 cm^−1^ and 150 consecutive readings to improve the signal–noise ratio.

## Results

### Tomb typology

**Figure 2:**
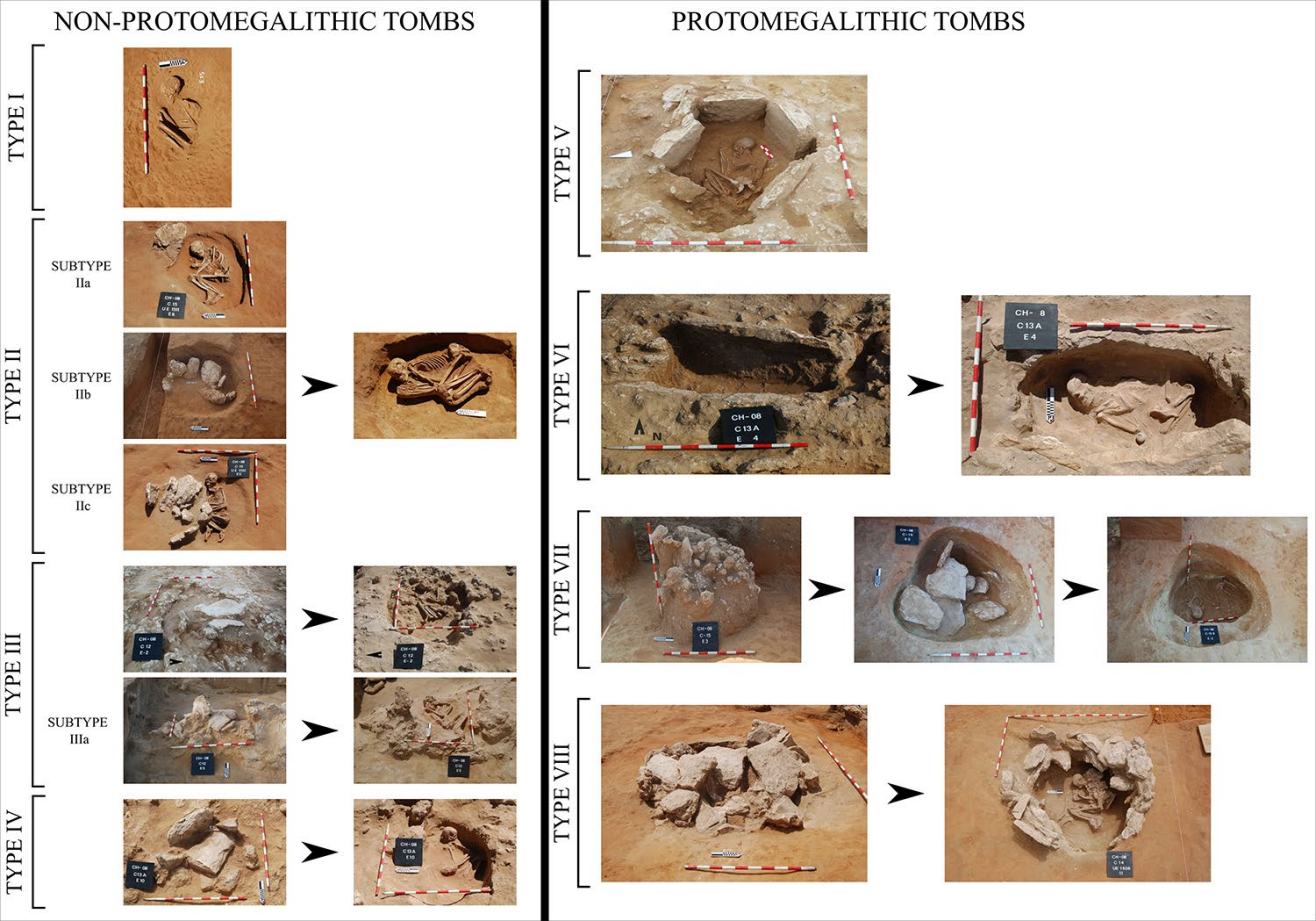
Typology of funerary structures at the Campo de Hockey necropolis. To the left, typology of non proto-megalithic tombs, and to the right typology of proto-megalithic tombs, in order of complexity.

The following types of funerary structures were established, based on constructive criteria (such as the use of pits and stone elements) (Fig. 2). We cannot rule out that some of the structures were sealed with organic material (hides, timber).

- ***Type I.*** Simple oval pit.
- ***Type II.*** Oval pit, of varying dimensions, outlined by stone slabs and uncovered. This type was subdivided into three subtypes, according to the complexity of the structure:
  - **Subtype IIa.** Pit with middle-size stone markers at the head or the feet.
  - **Subtype IIb.** Pit outlined all around with middle-size stone slabs.
  - **Subtype IIc.** Pit whose lateral sides are outlined by stone slabs placed at 45° angles to protect the pit and as markers.
- ***Type III.*** Oval pit covered by stone slabs horizontally placed as lids.
  - **Subtype IIIa.** Pit with horizontal stone slab lids and a vertical slab as a marker.
- ***Type IV.*** Pit whose perimeter is outlined with stone slabs, covered by a large stone slab and a vertical marker.
- ***Type V.*** Polygonal funerary chamber dug in the ground and outlined by six large stone slabs which, based on size and regularity, can be regarded as orthostates.
- ***Type VI.*** Pit dug into a platform formed by the geological substratum, closed on its northern side by two large stone slabs (orthostates), set at a 45° angle, signalling and protecting the burial.
- ***Type VII.*** Pit dug into the geological substratum. The burial is at the base, above which the cover (large size stone slabs) sits on a raised step. The structure is covered by a mound formed by large and middle size stones.
- ***Type VIII.*** Circular pit, 2 m in diameter, dug into the geological substratum. The funerary chamber is covered by a mound formed by superposed large stone slabs. The use of this amount of stone results in a monumental feature. The chamber is outlined by a perimeter ditch, approximately 1.15 m wide and 10 m in diameter.

### Radiocarbon dating: Bayesian analysis

**Figure 3:**
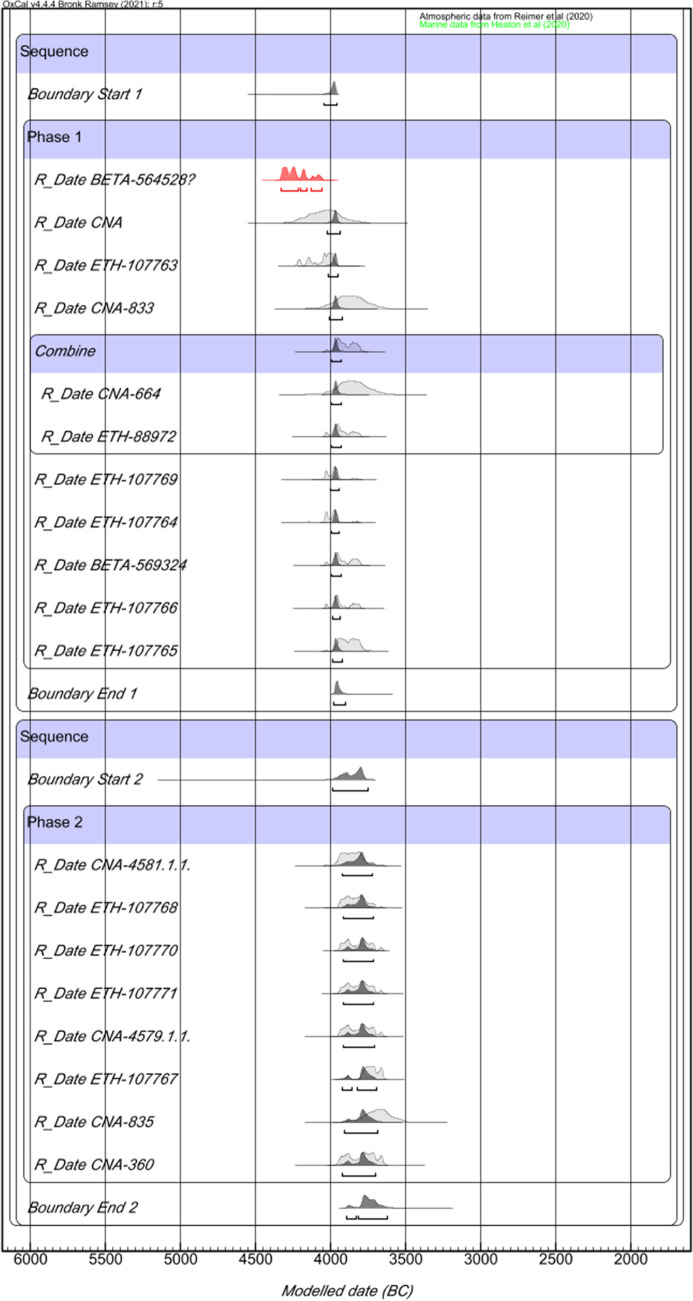
Overlapping Phases, Bayesian Modelling of radiocarbon dates from Campo de Hockey.

**Figure 4:**
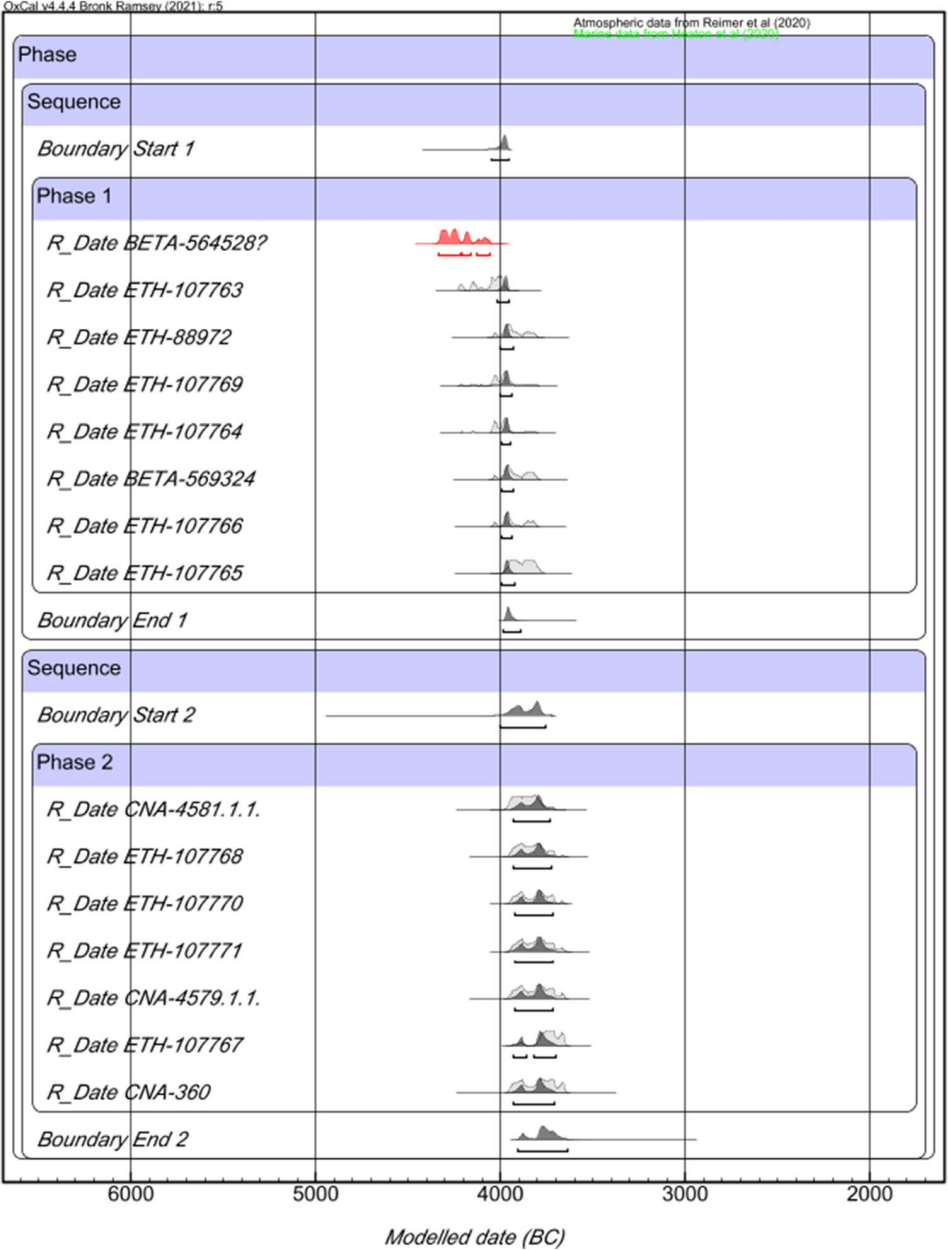
Overlapping Phases, Bayesian Modelling of radiocarbon dates obtained from human bone samples.

Two different Bayesian Models (Figs. 3 and 4, respectively) were used to determine the chronology of the site: the first one modelled all the available radiocarbon dates (Model 1), and the second only the human bone samples from burial contexts (Model 2). The aim was to establish whether there are chronological differences between the different kind of contexts found in the site and whether the radiocarbon dates from marine shells distorted the results by yielding excessively early or late dates.

Both models yielded virtually identical results, suggesting a continuous occupation of Campo de Hockey during two overlapping phases. Model 1 (Fig. 3. *Amodel.* 121 and *Aoverall.* 111.4) yielded a chronology between 4050–3960/3985–3900 cal BC (Phase 1: span of 0–100 years) and 3990–3755/3895–3620 cal BC (Phase 2: span of 0–215 years), while Model 2 (Fig. 4. *Amodel.* 135 and *Aoverall.* 126.8) yielded a chronology between 4050–3960/3985–3890 cal BC (Phase 1: span of 0–95 years) and 4000–3755/3900–3635 cal BC (Phase 2: span of 0–200 years).

Both models regarded the oldest radiocarbon date (BETA-564528) from E3T15 burial as an outlier, since its probability intervals (4335–4070 cal BC) are too old to belong to the first chronological phase. The radiocarbon date from this grave is contemporary to the dated seashell from the hearth T17/UE1712 structure (4315–4050 cal BC *Acomb. 64.8*). As such, the E3T15 burial and the hearth T17/UE1712 are the earliest dated evidence from the site. However, the regular occupation of the site did not begin until 4050–3960/3985–3900 cal BC (interval within which most of the earliest dates cluster).

**Figure 5:**
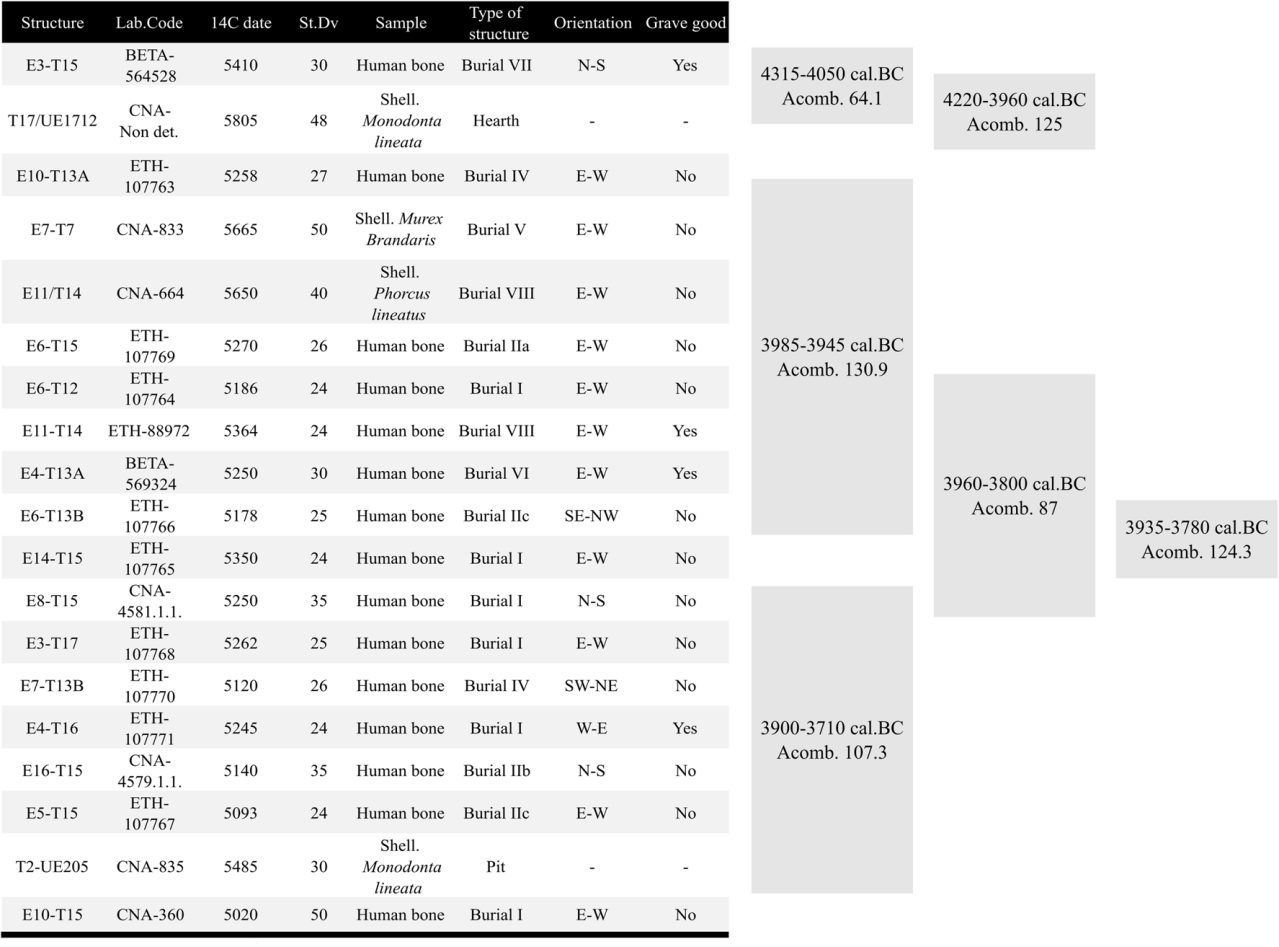
Synchronous contexts and patterns at Campo de Hockey, as defined by the Chi-square test of radiocarbon dates. Note: the letter C used in the column ‘Structure and individual’ in ^14^C corresponds to T in this paper (this is due to the Spanish–English translation of ‘trench’).

Finally, the Chi-Square test (*OxCal Combine* function) also was used to establish the degree of synchronicity between structures, patterns and individuals. The results suggest that, although not all of them were contemporaneous, some synchronous subgroups can be found (Fig. 5). These subgroups correspond to a particular spatial distribution of the site, the oldest (Burial E3T15 and Hearth T17/UE1712) and earliest (Burials E5T15 and E10T15) contexts being concentrated in the eastern sector, except for the pit found in trench 2 (UE 205), which, as noted, was located to the west, and thus farthest from the cemetery. The remaining dated burials are distributed between the eastern and the south-eastern sectors, and no spatial-chronological pattern can be attested. The only structure that does not follow this pattern is burial E7T7 (Phase 1), the sole documented structure in trench 7, in the westernmost sector of the site.

Regarding the different types of funerary structures, the Chi-Square test results suggest an interesting pattern that dates the proto-megalithic funerary structures (Types VIII, VII, VI and V) to the earliest half of the occupation of the necropolis, while Types I and II are spread throughout the second half. One of the Type IV graves is dated to the first half in the life of the necropolis, and the other to the second half.

A similar trend was documented regarding the orientation of buried individuals. An east orientation was the most common in the necropolis and the only one found in all proto-megalithic graves, and was especially prevalent during the first half of the site’s chronology (3985–3945 cal BC *Acomb. 130.9*). According to the test, no temporal correlations can be established regarding the presence/absence of grave goods. Burials with and without goods are documented throughout the entire occupation of the cemetery, suggesting different kinds of funerary expressions and practices within the community. However, there is a significant chronological overlap in the presence of non-local materials and proto-megalithic structures. All three amber objects found were located in this kind of burial (E3T15 and E11T14), dated to the earliest period in the occupation of the site. The green necklace beads are also related to the proto-megalithic burials, as two out of the three specimens documented were found inside this type of grave (E3T15); the third one was found in a non-adult Type I inhumation (E2T13B), that remains undated. Finally, the sillimanite axes are linked to Phase 1, and both polished stone tools were found in proto-megalithic graves (E11T14 and E3T15).

### Bioarchaeology

Out of 72 individuals analysed, 54 were identified as adults and 18 as non-adults, including 13 Infants I (18.1%); 5 Infants II (6.9%); 48 Adults (66.7%); and 6 matures (8.3%) (Table 3). By sex, 9 females (7 adults and 2 matures) and 15 males (12 adults and 3 matures) could be identified. The proportion of individuals whose sex could not be determined is unusual because of the poor state of preservation of the assemblage (Table 4).

**Table 3:** Distribution by age group.

Non adults			
Age range	Nº individuals	%	Total of individuals
Neonate	0	0	18
Infant I	13	18.1	
Infant II	5	6.9	
Juvenile	0	0	

Adults			
Age range	Nº individuals	%	Total of individuals
Adult	48	66.7	54
Mature	6	8.3	
Senile	0	0	
Total			72

**Table 4:** Distribution of adult individuals by sex and age.

	Female	Male	Indeterminable	Total
Adult	7	12	29	48
Mature	2	3	1	6
Total				54

**Figure 6:**
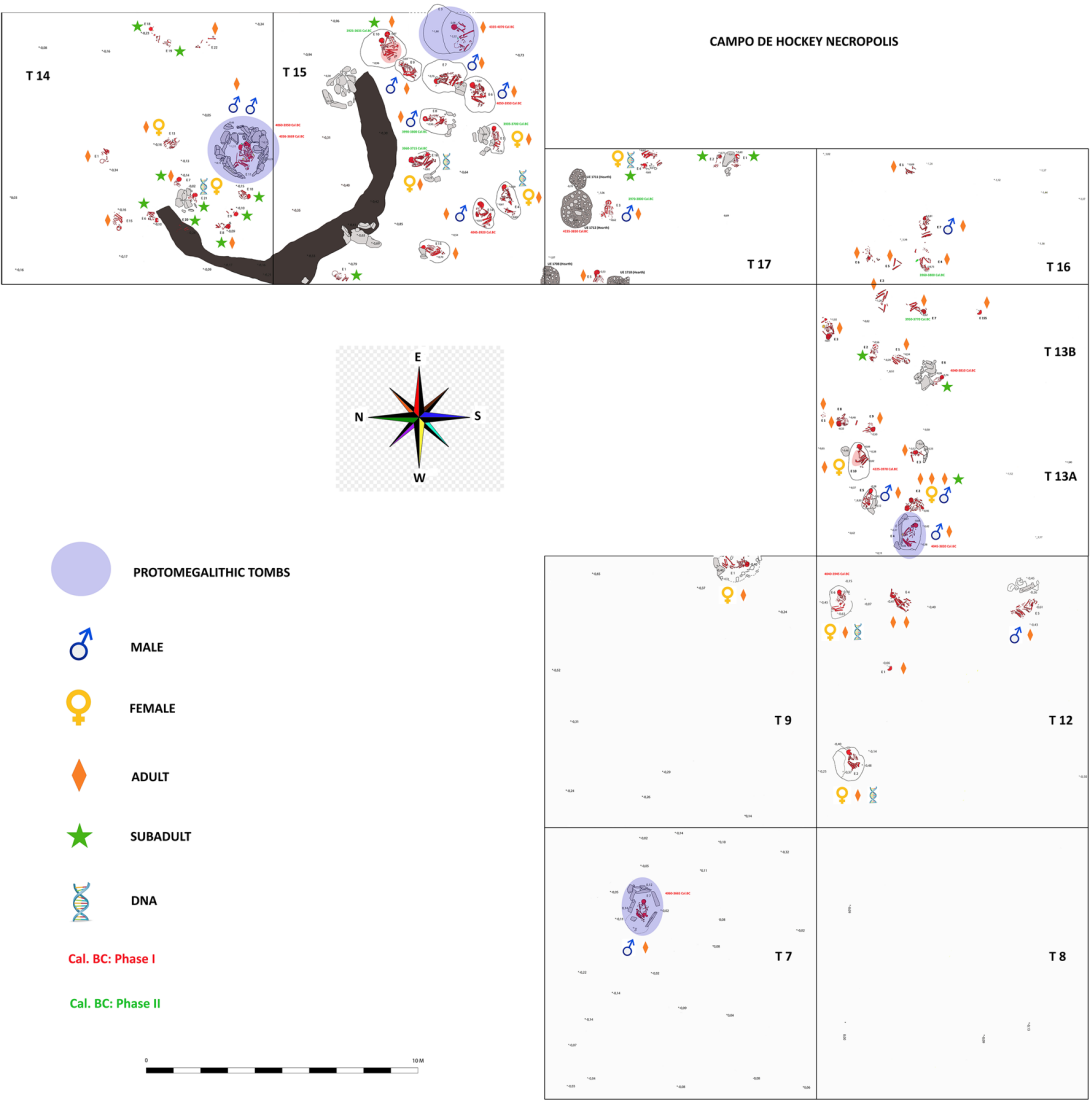
Plan of the Campo de Hockey necropolis, with indication of proto-megalithic tombs, sex, age, ADN-characterised individuals and radiocarbon dated tombs. Photo montage was designed with the application of Adobe Photoshop version CC 2021 (22.3.1) (http://www.adobe.com).

**Figure 7:**
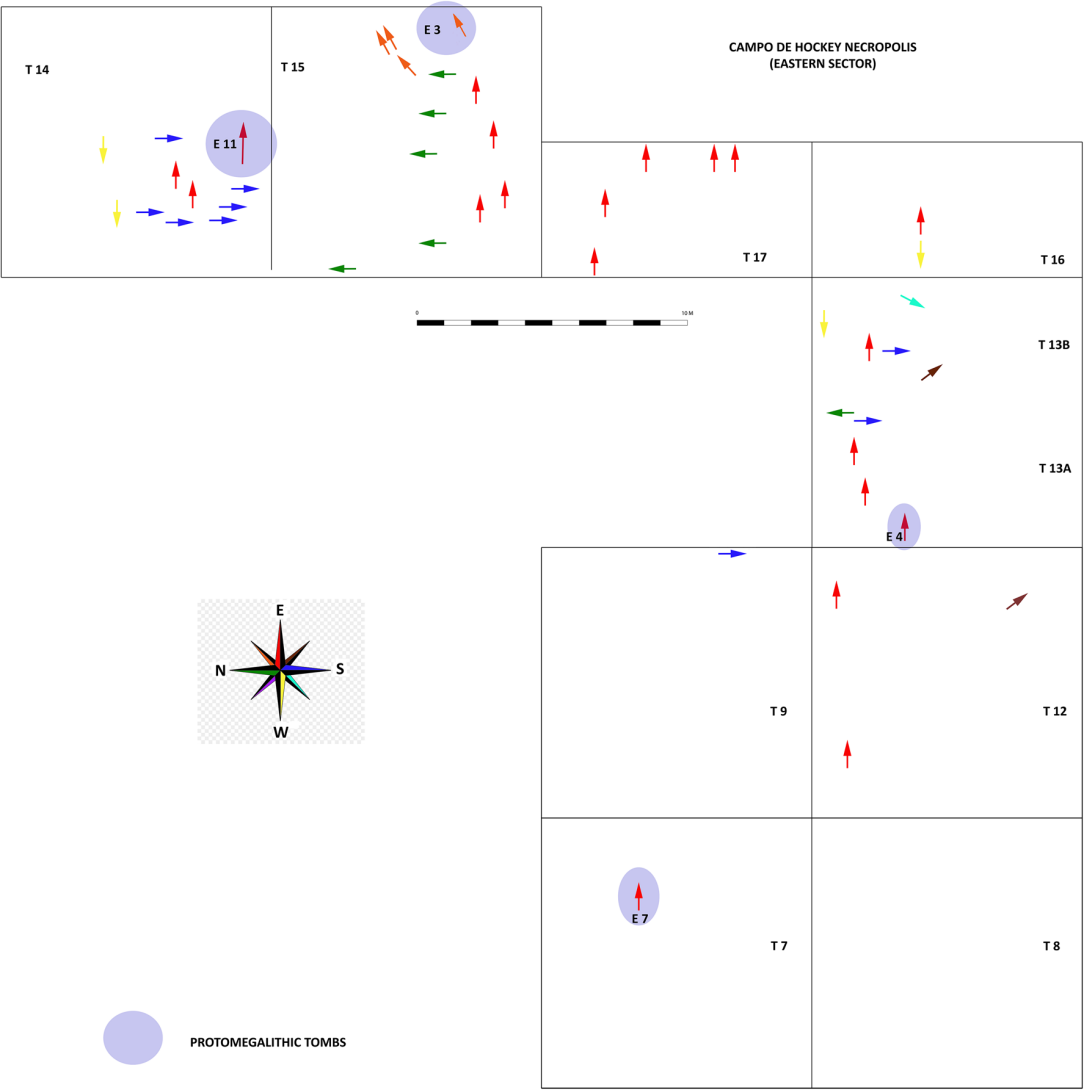
Plan of the Campo de Hockey necropolis, with indication of orientation of the individuals (the arrowhead would indicate the position of the head). Photo montage was designed with the application of Adobe Photoshop version CC 2021 (22.3.1) (http://www.adobe.com).

Concerning the type of burial, the 53 graves excavated include 45 single inhumations in which the individual is placed in foetal position or on one side, and several collective burials, including seven double and one quadruple inhumations (Supplementary Table S1 online). Regarding the spatial distribution of individuals by sex, it is worth mentioning that proto-megalithic tombs were exclusively used for males. In terms of age, the large proportion of infantile individuals towards the north of monumental burial E11T14 is to be emphasised (Fig. 6). Similarly, the orientation of the 46 articulated individuals could be determined: 20 individuals were buried facing E-W; 9 S-N; 6 N-S; 4 W-E; 4 NE-SW; 2 SE-NW; and 1 SW-NE (Fig. 7).

### DNA

**Figure 8:**
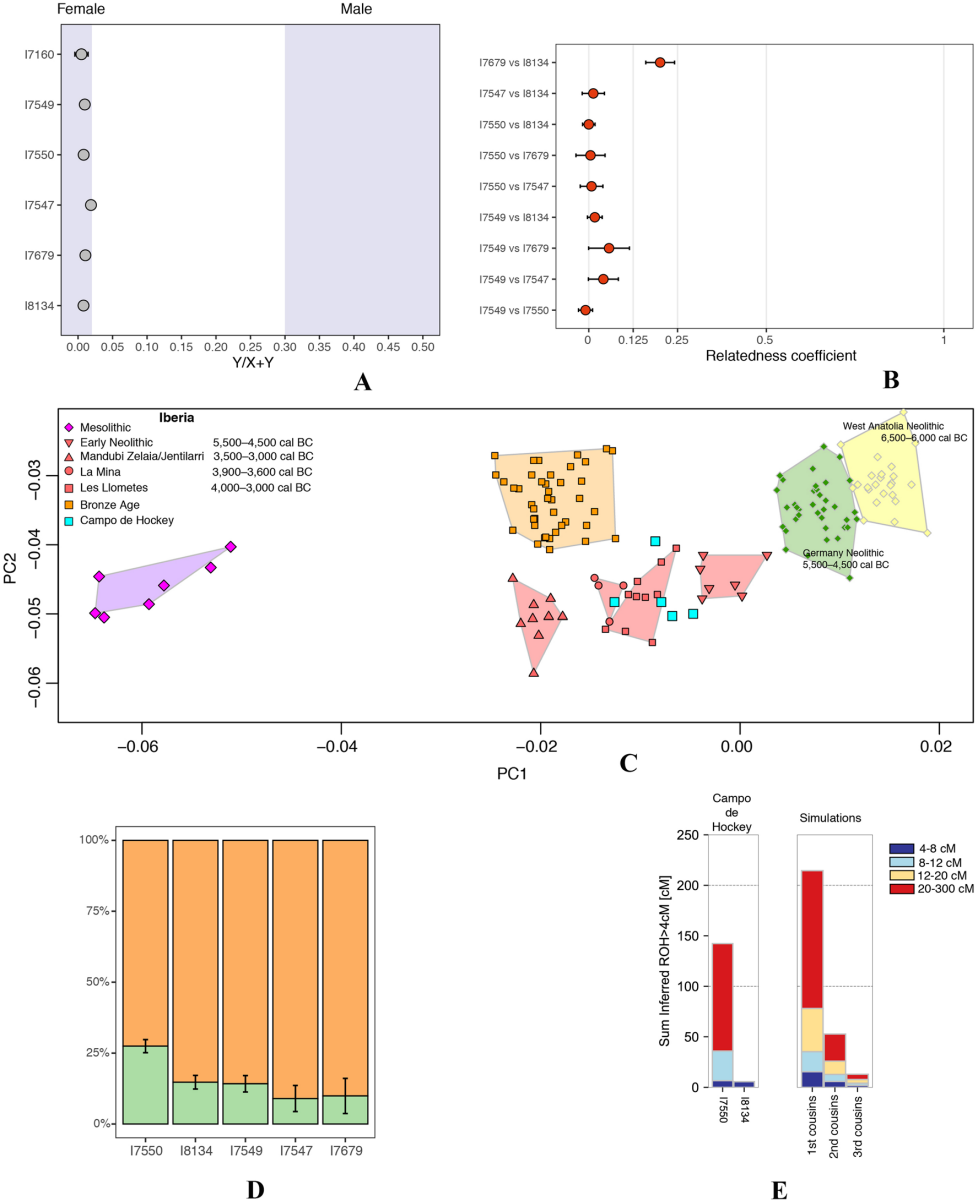
(**A**) Genetic sex determination using the ratio of the number of DNA sequences mapping to the Y-chromosome and the total number of sequences mapping to sex chromosomes^37^. Bars represent 95% confidence intervals. (**B**) Relatedness coefficients computed on the autosomal chromosomes as in ^36^ for all the pairwise comparisons between Campo de Hockey individuals. Bars represent 95% confidence intervals. (**C**) Principal Components Analysis of Campo de Hockey individuals and other ancient individuals from Iberia and other European populations. Using *smartpca* in *Eigensoft* software^40^, ancient individuals were projected onto the principal components computed on a set of present-day West Eurasians (not shown in the figure) genotyped on the Human Origins Array. (**D**) Ancestry proportions deriving from Iberian Early Neolithic populations (orange) and from European Mesolithic hunter-gatherers (light green), computed with *qpAdm.* Bars represent ±1 standard error. (**E**) Runs of homozygosity length distribution computed with hapROH^45^.

The six individuals under study were females according to the genetic analysis (Fig. 8A). Regarding kinship, these six individuals shared no mitochondrial lineage (Table 5), ruling out a recent common maternal ancestor. On the autosomes, only one close kin relationship was identified between individuals I7679 and I8134, who shared 20% of their genomes and are thus 2nd- or 3rd-degree relatives (Fig. 8B). If they were 2nd degree relatives, given that they have different mitochondrial lineages (ruling out maternal half-sisters, niece-maternal aunt and granddaughter-maternal grandmother) and that they do not share DNA in the X-chromosome (ruling out paternal half-sisters, and granddaughter-paternal grandmother), they could only be a niece and her paternal aunt (order unknown).

**Table 5:** Summary of genetic results.

ID	Skeletal code	Skeletal element	Damage rate in first nucleotide on sequences overlapping 1240 k targets	endogenous DNA (%)	mtDNA coverage	mtDNA haplogroup	mtDNA match to consensus	Coverage on autosomal targets	SNPs hit on autosomal targets	Genetic Sex	Sum total of ROH segments > 4 cM	Sum total of ROH segments > 20 cM
I7160	CH-08-T15-UE1514-E16	Tooth	0.086	0.5	2.17	T2c1d	[0.938, 1.000]	0.004817	5691	F	n/a (too few SNPs)	n/a (too few SNPs)
I7549	CH-08-T12-UE1214-E6	Petrous	0.176	2.6	34.9	K1a4a1	[0.991, 1.000]	0.285374	263,719	F	n/a (too few SNPs)	n/a (too few SNPs)
I7550	CH-08-T15-UE1502-E4	Petrous	0.182	7.6	49.8	K1b1a	[0.986, 0.998]	1.185428	637,799	F	142.306493	106.38619
I7547	CH-08-T12-UE1210-E2	Petrous	0.099	1.7	17.6	K1a + 195	[0.942, 0.976]	0.058102	65,130	F	n/a (too few SNPs)	n/a (too few SNPs)
I7679	CH-08-T14A-UE1402-E21	Petrous	0.158	0.5	4.56	H	[0.943, 1.000]	0.030499	35,149	F	n/a (too few SNPs)	n/a (too few SNPs)
I8134	CH-08-T17A-UE1709-E4	Petrous	0.23	.	56	U5b3	[0.965, 0.986]	1.673109	662,946	F	5.6782	0

Regarding ancestry, the Campo de Hockey individuals fall within the genetic variation of Iberian populations between 4500–3000 BC. During this period, there is a geographical gradient of affinity to European Mesolithic hunter-gatherers^39^, with southern groups such as Llometes (Alicante, Spain) showing less affinity (higher values in PC1) and northern groups such as Mandubi Zelaia/Jentillarri (Gipuzkoa, Spain) showing more affinity (smaller values in PC1). In accordance to their geographic location, individuals from Campo de Hockey are closer to the Llometes individuals. Interestingly, female I7550 is shifted from the other individuals from Campo de Hockey in the direction of European Mesolithic hunter-gatherers (Fig. 8C), suggesting ancestry heterogeneity at the site. We formally tested whether this individual shared significantly more alleles with Mesolithic hunter-gatherers as compared to the remaining individuals from the site, using the f4 (Mbuti, Western hunter-gatherers; Campo de Hockey, I7550). This statistic was significantly greater than 0 (Z-score = 4.19) indicating a greater affinity of I7550 for Mesolithic hunter-gatherers and confirming the observation in PCA. Finally, we estimated for each individual the proportions of ancestry deriving from Iberian Early Neolithic populations and from European Mesolithic hunter-gatherers (Fig. 8D). Female I7550 derives 27% of her ancestry from European Mesolithic groups, while the other individuals present 9–15% of this type of ancestry.

Female I8134 did not show any long ROH, while female I7550 clearly show long ROHs (Fig. 8E) compatible with a first cousin relation of her parents. We obtained pigmentation predictions for the three individuals with higher quality data, which indicated highest probabilities for brown eyes, black hair and intermediate skin colour (Table 6).

**Table 6:** Likelihood of eye, hair and skin pigmentation phenotypes under the HIrisPlex-S model.

Sampleid	PBlueEye	PIntermediateEye	PBrownEye	PBlondHair	PBrownHair	PRedHair	PBlackHair	PLightHair	PDarkHair	PVeryPaleSkin	PPaleSkin	PIntermediateSkin	PDarkSkin	PDarktoBlackSkin
I7160	NA	NA	NA	NA	NA	NA	NA	NA	NA	NA	NA	NA	NA	NA
I7549	0.00126969	0.02653689	0.97219342	NA	NA	NA	NA	0.32167332	0.67832668	NA	NA	NA	NA	NA
I7550	1.29E−05	0.00209115	0.99789593	0.00298293	0.07978214	0.00088155	0.91635338	0.01004992	0.98995008	0.00487574	0.047715	0.94740.895	9.77E−14	3.10E−07
I7547	NA	NA	NA	NA	NA	NA	NA	NA	NA	NA	NA	NA	NA	NA
I7679	NA	NA	NA	NA	NA	NA	NA	NA	NA	NA	NA	NA	NA	NA
I8134	1.65E−05	0.00231.427	0.9976692	0.00448681	0.11655496	0.00089021	0.87806803	0.03150238	0.96849762	3.39E−10	1.61E−11	1	1.65E−14	8.76E−10

### Grave goods

**Figure 9:**
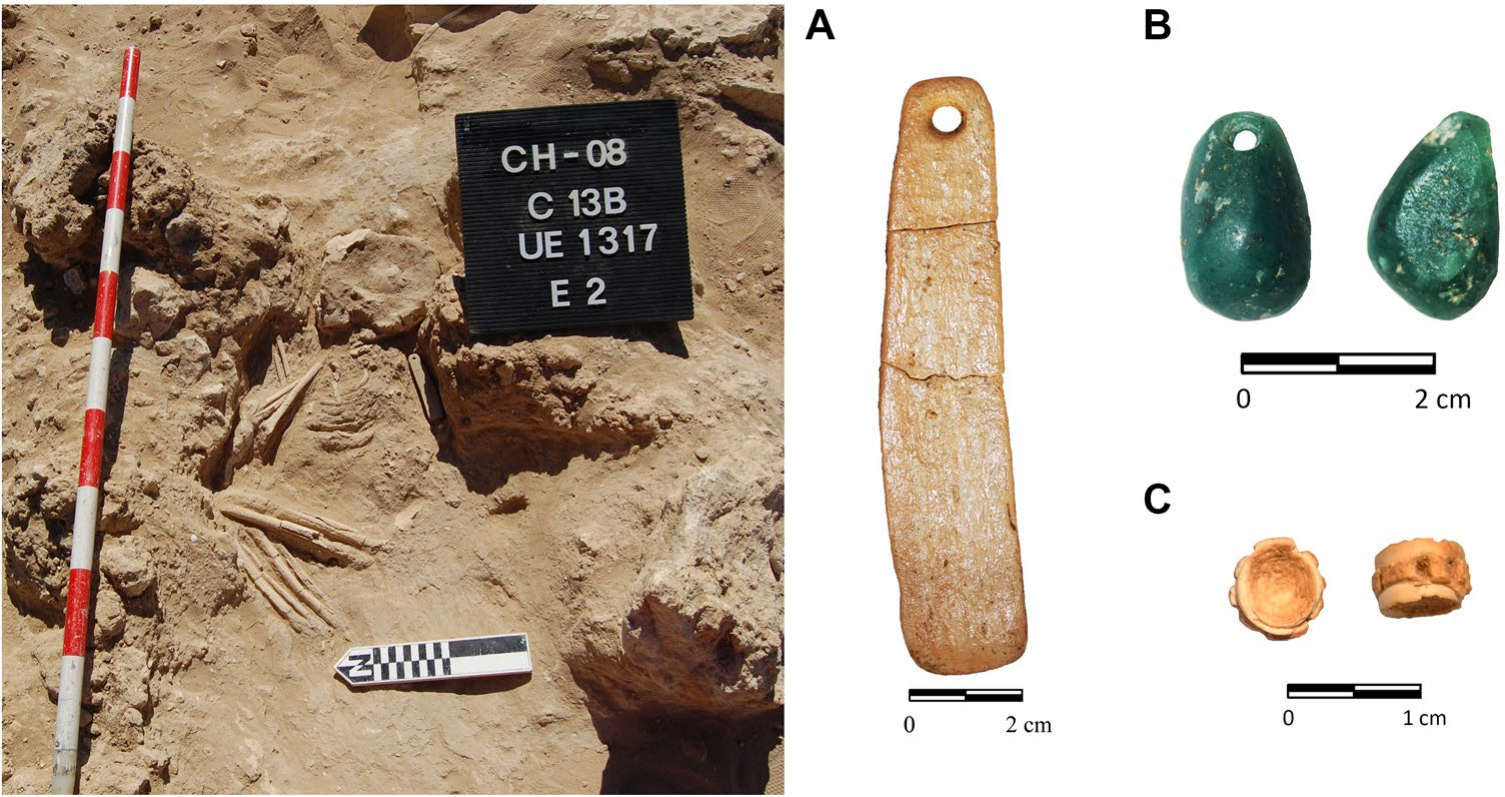
Infant burial E2T13B and grave goods, consisting of a bone pendant with a bevelled edge (**A**), a green mica pendant (**B**), and a possible pendant carved on a elasmobranch vertebra (**C**).

**Figure 10:**
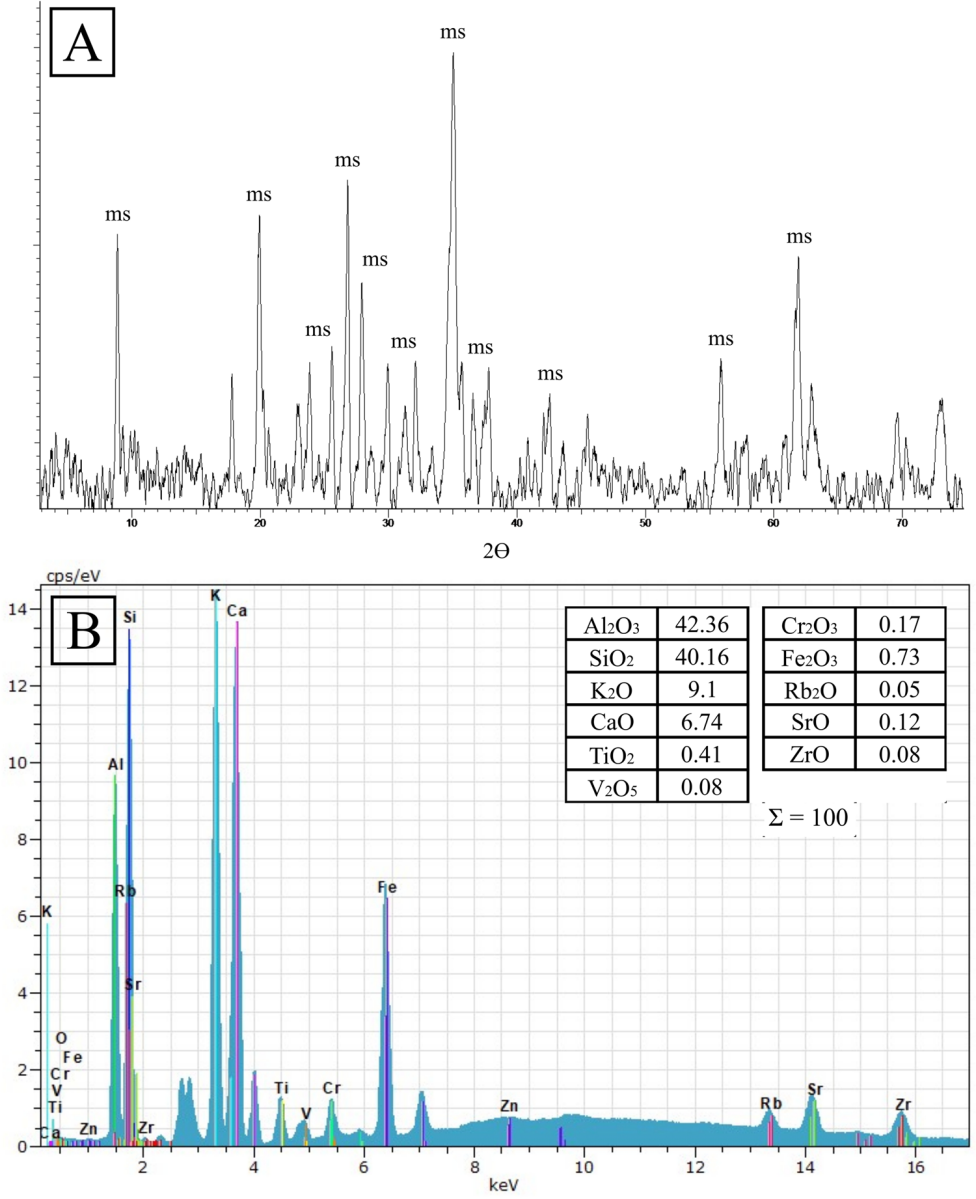
(**A**): X-ray diffractogram X (Ms: muscovite) and (**B**) X-ray microfluorescense spectrum from the surface of the green bead from E2T13B, with quantitative chemical analysis by mXRF (oxides percentages).

Some of the burials at Campo de Hockey yielded grave goods that can be considered as exotic, that is, organic or inorganic materials that are scarce, rare or foreign to the region^17^. These items, which have a clear social and prestige component were not of practical nature^50^ and this includes exotic polished stone objects, such as axes and adzes in sillimanite (*var*. fibrolite) or other metamorphic or volcanic rocks^17^. The source of some of these materials is often far away, and their discovery in southern Europe during the Middle Neolithic reveals the operation of prestige goods distribution networks, which often included sea routes^50–56^. In fact, the oldest grave (E3T15, Type VII) yielded two beads carved in green stone (variscite and turquoise), previously analysed by Vijande et al.^17^. The cleaning of bone remains that preceded scientific analysis led to the identification of a third bead carved in green stone, from an infant burial (E2T13B). This unpublished oval tear-shaped pendant (Fig. 9) was analysed by X-ray diffraction and X-ray fluorescence, which identified it as a mica-type silicate mineral; the diffractogram presented the typical reflections of muscovite 2M and margarite 2M (Fig. 10A). The granular texture and intense green colour, probably resulting from the high content in such elements as Cr, which were also detected by mXRF, should be highlighted (Fig. 10B).

**Figure 11:**
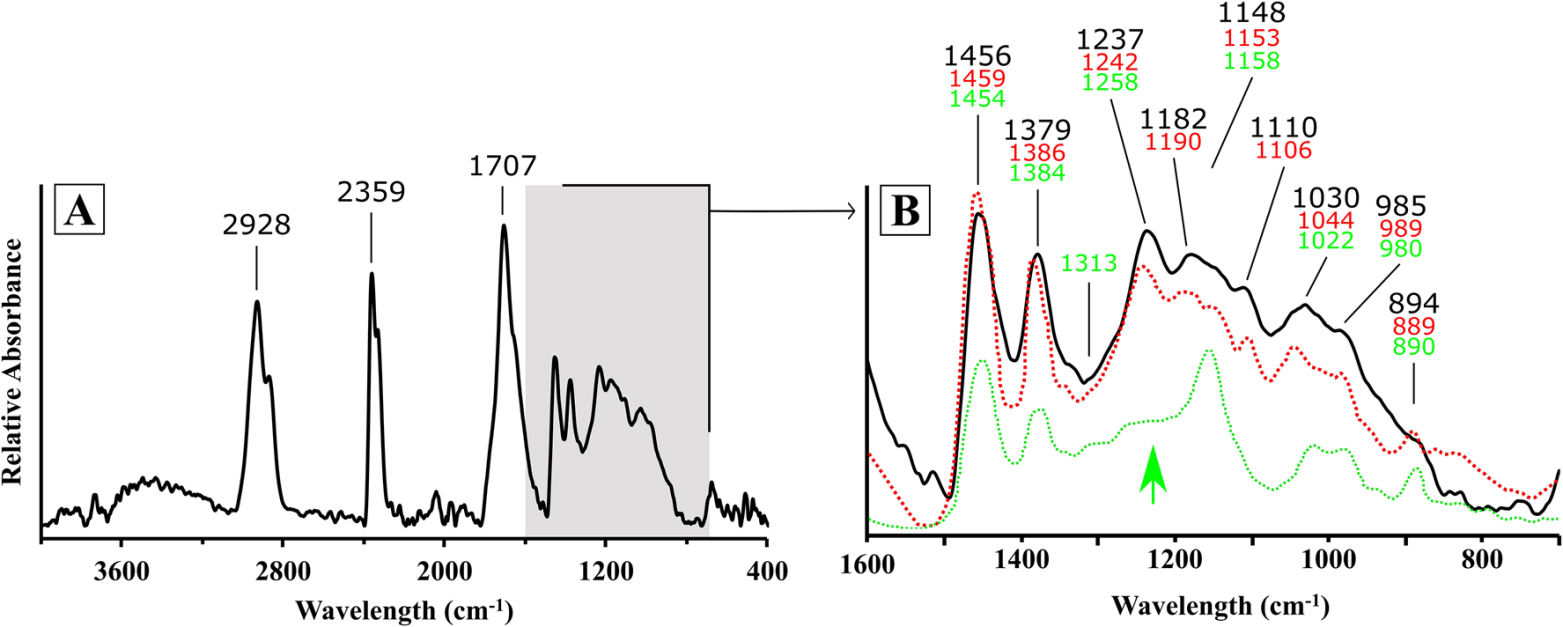
FTIR spectrum of the unaltered inner face of sample CH VE1405 (**A**) and detail of grey area (**B**) indicated by black line. The red dotted line shows the pattern of simetite and the green dotted line that of succinite, based on ^55^. The green arrow indicates the approximate location of the ‘Baltic shoulder’.

All three amber pendants identified were found in proto-megalithic burials (one of them in situ, on the neck of the individual buried in E4T13A, Type VI; another in burial E3T15, Type VII; and another in burial E11T14, Type VIII)^17^. The three pendants are dark reddish brown in colour, and their surface is heavily altered, which hampered comparison with known geological patterns in the Iberian Peninsula and elsewhere. Preliminary analysis by FTIR confirmed that the samples are not Baltic in origin^17^, as they did not present the typical “Baltic shoulder” at 1250–1110 cm^−1^, followed by a well-defined band at 1157 cm^−1^ (e.g. ^57^). For this reason, further FTIR analyses were undertaken, and the results compared with Sicilian, Polish, Spanish and Portuguese patterns, from the literature and in the project’s own reference collection^58–60^. The resulting spectrum is illustrated in Fig. 11A, and the characteristic bands are annotated. Figure 11B illustrates the grey area in Fig. 11A in detail to facilitate comparison with Baltic (succinite) and Simeto (simetite) amber patterns published by Devièse et al.^58^. The spectrum on the sample (continuous black line) fits well with the simetite sample (red dotted line) and lacks the Baltic shoulder visible in the succinite sample (green arrow and green dotted line). Finally, non-exotic grave goods documented in Campo de Hockey (bone hairpins; ceramic vases; bone pendants; ochre) were published in earlier works^17^.

Ochre, identified as hematite, was found in two graves (E10T13A and E10T15). Total mercury (THg) concentrations were estimated in eighteen burials from Campo de Hockey using inner cortical bone from the shaft of the humerus, femur, and tibia (see methodology in^61^). Although high concentrations of THg in human bone from late Neolithic to Chalcolithic sites in Iberia are often linked with mining and the use of cinnabar (HgS^61^), no evidence of this mineral was found in any of the burials at Campo de Hockey. Thus, the values of THg recorded here are likely the result of environmental background and dietary exposure.

**Figure 12:**
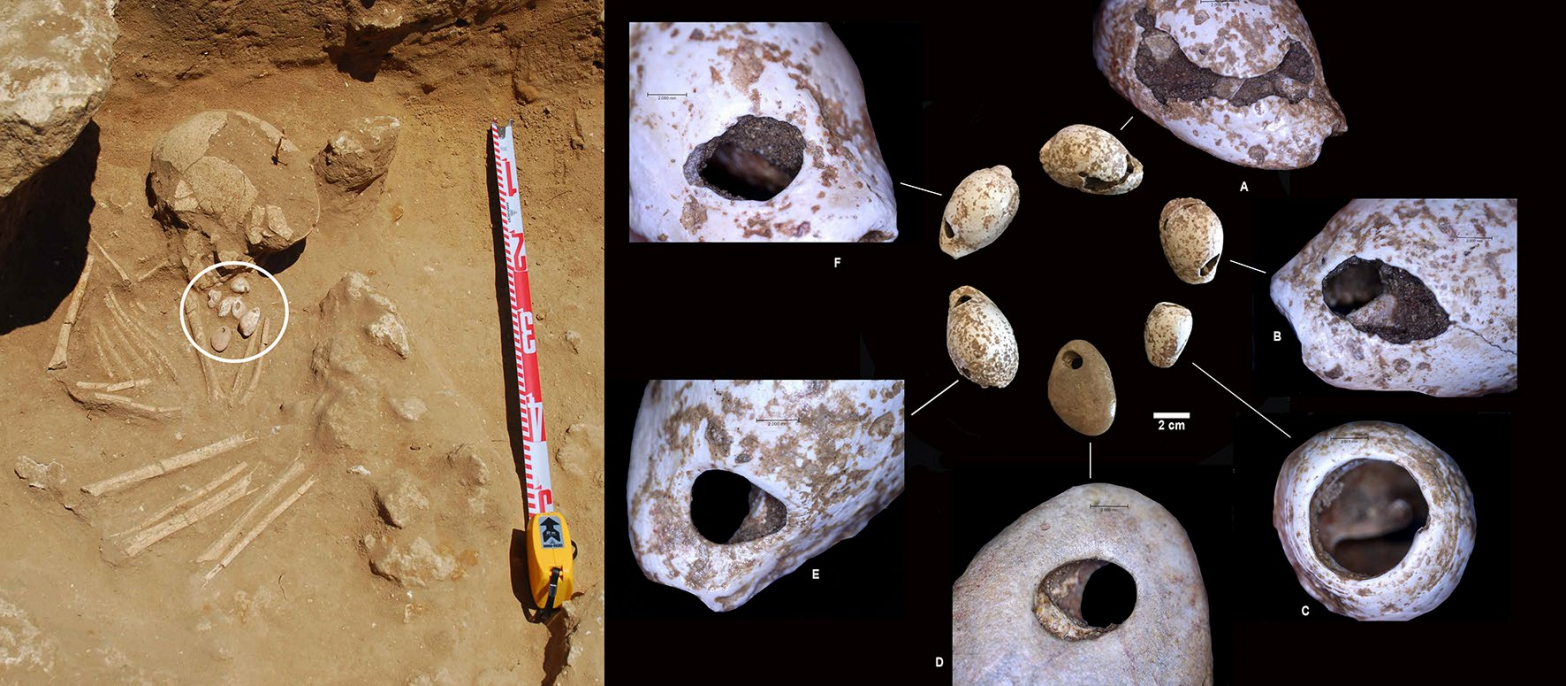
To the left, infant burial E1T17, where a shell necklace was found in situ, near the neck area. To the right, detail of the necklace, formed by four specimens of *Zonaria Pyrum* (**A**,**B**,**E**,**F**); one of *Conus mediterraneus* (**C**); and a flat beach pebble (**D**).

The importance of the sea for this community is reflected in the presence of numerous items of a marine origin in the graves. Especially of note is the presence of full malacological items situated next to the head of several individuals^20^. Also significant is a necklace made of five seashells and a sea pebble (Fig. 12D) around the neck of the child buried in grave E1T17. Four of the shells belong to *Zonaria pyrum* (Fig. 12A,B,E,F) and the one to *Conus mediterraneus* (Fig. 12C). All of them are perforated deliberately, some by percussion/pressure (Fig. 12A,B) and some by friction (Fig. 12E,F). Single (Fig. 12F) and double perforations are attested (Fig. 12A,B,E,F). In the specimen of *Conus mediterraneus*, the perforation was achieved by the removal of the apex (Fig. 12C). These personal adornments were likely bound by an element made with organic material that has not survived. However, their primary position over the body has provided valuable information concerning the shape, arrangement and characteristics of the necklace; this is a rare example of archaeological necklace found in situ*.* Finally, the excavation of infant grave E2T13B yielded a rare bead carved in a ray vertebra (Fig. 9).

## Discussion and conclusions

Traditionally, the origin of Megalithism has been associated with a proto-megalithic stage characterised by small stone monuments, consisting of single-period corridor-less chambers. The main problem in our case is that few proto-megalithic necropolises in the Iberian Peninsula have been reliably dated (short-life samples). Campo de Hockey is one of the rare exceptions, hence the site’s importance for understanding the origins of Iberian funerary Megalithism. The 19 radiocarbon dates from Campo de Hockey (15 of which were obtained from human bone) have enabled the application of a valuable Bayesian model. The chronological analysis has outlined two overlapping phases: 4050/3960/3985–3900 cal BC (0–100 year span) and 3990–3755/3895–3620 cal BC (0–215 year span). These results confirmed the site’s continuous occupation over several generations (around 300 years). Grave E3T15 is an outlier, yielding a slightly earlier date, and thus representing the first occupation of the site between 4335–4060 cal BC.

The Bayesian analysis characterises Campo de Hockey as one of the earliest proto-megalithic necropolises in the Iberian Peninsula. Similar chronologies are found in Tomba del Segudet (Andorra), in which a human bone sample was dated to 4327–4051 cal BC 2σ^62^, in what is the earliest date for a proto-megalithic structure in the NE of the Iberian Peninsula^63^. The NW has been regarded as one of the source areas of proto-megalithism in the Iberian Peninsula. The earliest megalithic structures found in the region include small polygonal chambers or mound-covered pit burials. A chronology in the late 5th millennium BC has been suggested for these structures^64,65^. Due to the poor state of preservation of the bone remains, this chronology was based on thermoluminescence and carbon dates from carbon samples found in problematic contexts, such as paleosols and debris from the interior of the mounds^66^. The earliest bone-based radiocarbon dates are from the Dolmen de Areita 1, in the Portuguese region of Beira. The dates—4840 ± 60 (GrA-18497) and 5170 ± 60 (GrA 18518), calibrated by OxCal 4.4 yield 3770–3383 (95.4%) and 4226–3796 (95.4%) respectively—situate these graves in the late 5th and early 4th millennium BC. Similar dates have been obtained in the Dolmen de Cotogrande 1 (GrA 18420: 4940 ± 80, calibrated to 3952–3535 cal BC, 95.4%), and Chan da Cruz 1 (CSIC-642: 5210 ± 50, calibrated to 4233–3947 cal BC, 95.4%), both in Galicia; these dates were obtained from carbon and organic residue respectively^12,65,66^. Therefore, the data available do not support that megalithism began in the NW of the Iberian Peninsula before the turn of the 4th millennium BC. Finally, dates obtained in Cantabria, which present a deviation of under 100 years, point to the second quarter of the 4th millennium BC for the beginning of funerary megalithism in the region^67^.

Recent radiocarbon dates situate the origins of megalithism in southern Portugal after 3700 BC, challenging earlier ideas regarding the date of the beginning of this phenomenon in the region to the early 5th millennium BC^68,69^. In Andalusia, the earliest dates correspond to the necropolis of Arroyo Saladillo (Antequera, Málaga), in which a single proto-megalithic burial (structure 94) was dated to 4041–3803 cal BC 2σ^70^. This is the only stone-built grave in the entire necropolis. This is an oval mound, 2.6 m in diameter, made with large stones. Under the mound, two large stone slabs were found to cover a pit containing a single burial and modest grave goods^70^. In the province of Cádiz, the earliest funerary megalith was the Dolmen de Alberite I (Villamartín), dated to the turn of the 4th millenium; this chronology, however, was obtained from a carbon sample that did not allow for greater precision^71^.

Necropolises characterised by pit graves began appearing in the Iberian Peninsula in the late 6th and early 5th millennium BP. Regional differences can be attested, however, for instance the emergence of stone objects in some of the graves. This phenomenon is well known in the north east of the Iberian Peninsula, with the so-called ‘pit graves culture’, recorded in such sites as Bòbila Madurell-Can Gambús^72,73^ and, in the middle Ebro valley, the necropolises of Los Cascajos and Paternanbidea^74,75^.

Similar in chronology or slightly later than Campo de Hockey, monumental graves are attested in the Tagus and Douro valleys: e.g. the stone cist at La Peña de La Abuela; the mound at Sima I, in the Ambrona valley^76,77^; and, the dolmen of Azután in Toledo^78^, one of the earliest megaliths in the Iberian Peninsula, along with the foci found in Portugal^68,79^, Galicia and the Grupo de Tavernet^66^. Outside the Iberian Peninsula, closed stone chamber structures^66^ are found in southern France and Northern Italy; parallels exist in Monte Revincu, Corsica^80^, which are similar to the protomegalithic constructions in Campo de Hockey.

**Figure 13:**
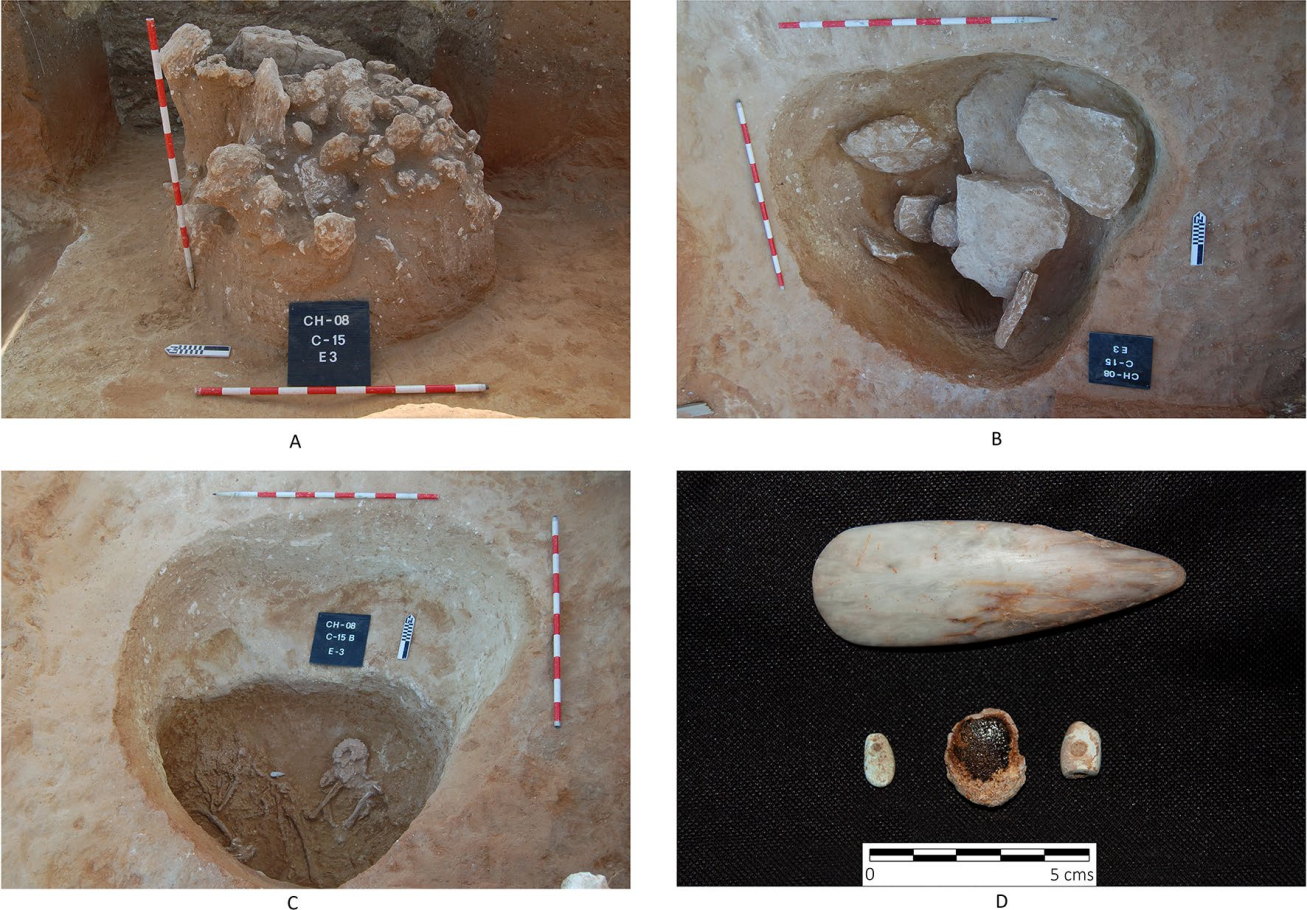
Burial E3C15 (Type VII) during excavation: (**A**) Stone mound that covered the feature; (**B**) Stone slab covers under the mound; (**C**) Burial level, with an individual on its right side; and, (**D**) grave goods, including a sillimanite axe; an amber pendant; a variscite bead; and a turquoise bead.

The earliest proto-megalithic burial in Campo de Hockey (E3T15) also stands out by the grave goods found in its interior: an amber pendant, a variscite bead, a turquoise bead and a small sillimanite axe (Fig. 13). These objects suggest the operation of prestige good distribution networks in the Iberian Peninsula from the late 5th millennium BC if not earlier. The Sicilian origin of the amber (simetite) and the fact that San Fernando was an island during this period, indicate that basic seafaring techniques were known^17^.

Another interesting fact is that the proto-megalithic graves are the earliest in the site; Types V, VI and VIII fall in Phase I, while Type VII (E3T15) even slightly predates this phase (Fig. 13). The radiocarbon determinations also yielded interesting patterns regarding the presence of non-local materials deposited as grave goods, especially concerning the link between the amber deposited during the site’s earliest period of occupation and proto-megalithic burials; this confirms the arrival of Sicilian amber to the Iberian Peninsula in the late fifth century BC^59,60,81^. Also interesting is the presence of variscite and turquoise in grave E3T15, demonstrating that these stones were used in the SW of the Iberian Peninsula earlier than previously thought^17^. The only sillimanite axes found in Campo de Hockey were found in the most monumental graves (Types VII and VIII)^17^.

Moreover, the contemporaneity between proto-megalithic graves (with exotic grave goods) and simple burials (without exotic grave goods), suggests social inequality within the community^17^. Phase II is characterised by the absence of monumental graves and prestige goods, which could be pointing to a period of decadence of the settlement.

Furthermore, the synchronicity between burial E3T15 and hearth T17/UE1712, as well as that between the second-phase of burials and pit T2-UE205, confirms that the community was building different types of features simultaneously. Moreover, the contemporaneity of seashell and human bone radiocarbon samples confirms that seashells used for funerary purposes were consumed shortly before the death of the inhumated individual.

The correlation between burial dates and other elements is also interesting, that is reflected in the spatial–temporal distribution patterns; while the earliest contexts concentrate in the eastern sector of the site, later features shift to the eastern and south-eastern sectors. Concerning the orientation of the burials, most individuals (52.17%) were placed facing east (E or NE) (Fig. 7), a tradition preserved in later megalithic constructions^70,82^. In addition, the number of individuals placed facing south or north is overrepresented in trenches 14 and 15, because in this area most graves were oriented using the main funerary structure (E11T14, Type VIII) as reference. It is important to point out that, as well as being the most monumental burial in the necropolis and hosting one of the most interesting grave good assemblages (1 amber pendant, 1 sillimanite axe and a ceramic vase), this grave contained the remains of two adult males buried at different times and presenting *perimortem* wounds in the skull^71^.

From an anthropological perspective, the proportion of subadult individuals identified is noteworthy (28%). This is unremarkable, as infant mortality in Neolithic communities must have been very high. The necropolis, however, confirmed that, at least in this community, subadult individuals were regarded as deserving burial treatment. In most cases, at any rate, subadults were buried in simple pits (fourteen in Type I graves and three in Type II graves) devoid of grave goods, with the exception of burial E2T13B, a Type I grave that yielded a bone pendant, a green mica pendant and a possible bead carved in an elasmobranch vertebra (Fig. 9), and burial E1T17, which yielded a seashell necklace (Fig. 12).

Of the eighteen identified subadult individuals, thirteen belong to the Infant I (6 months–7 years) age group, and five to the Infant II (7–14 years) age group, also an unremarkable circumstance as infant mortality must have been especially high during the early years. Although these graves proved that in this community infants were regarded as deserving of burial, the absence of neonates (0–6 months) is significant, since this should be the most numerous group. This absence could suggest that neonates were not regarded as deserving funeral attention, being considered socially inferior, or that they were disposed without leaving any archaeological trace. Most non-adult individuals were buried on their own in simple pit graves, although a significant proportion of their graves were found clustered to the north of E11T14, the most monumental grave in the necropolis. This could be caused by the existence of kinship ties between the two individuals found inside E11T14 and the subadult individuals buried north of them, or by ritual causes, whereby the subadult members of the community were buried in relation to its two foremost members. Concerning adults, it should be mentioned that only six fell in the middle-age group (41–60 years), and none in the advanced age group (≥ 61 years).

Regarding the sex distribution, that megalithic tombs were exclusively occupied by males is interesting (one male in grave E7T7-Type V; one in grave E4T13A-Type VI; two in grave E11T14-Type VIII; and one undetermined individual in grave E3T15-Type VII). Therefore, although these data suggest that monumental graves were restricted to males, more evidences are necessary to confirm this pattern. Despite the difficulties involved in obtaining DNA from such early remains in an open air site in the south of the Iberian Peninsula, results from six individuals were obtained, all of them being females (genetic sex matches anthropological sex)^39^. Although the Campo de Hockey settlement post-dates the earliest Neolithic in the region by over a millennium, female I7550 owes 27% of her ancestry to European Mesolithic groups, while the other individuals only present 9–15% of this type of ancestry. Previous studies^47,84–86^ have documented a resurgence of European Mesolithic-related ancestry in Iberia during the Middle Neolithic period, but Campo de Hockey is unique among other Iberian Neolithic sites in that it shows significantly different proportions of Mesolithic-related ancestry in individuals from the same site. This difference could indicate that the resurgence mentioned above was taking place during the period when the Campo de Hockey necropolis was in use, and that mixing between Neolithic groups and remaining pockets of indigenous hunter-gatherers, or perhaps between farmer groups with different levels of indigenous hunter-gatherer-related ancestry, was ongoing.

Concerning kinship, it was possible to determine that individuals I7679 and I8134 (which share 20% of their genome) were 2nd- or 3rd-degree relatives. Similarly, evidence for endogamy was detected in one individual. In fact, I7550 is the individual with the highest endogamy identified to date in the Iberian Peninsula, with the exception of a Magdalenian individual from El Mirón^87^. The fact that one individual (out of the two individuals in which this analysis could be performed) from Campo de Hockey was the daughter of two closely related parents could indicate that endogamy was a common practice in this community, either as a consequence of isolation from other groups (Cádiz was an island at the time) or of cultural reasons. Finally, the genetic data from three individuals suggest that their physical appearance was typical of Neolithic Mediterranean populations, with brown eyes, black hair and intermediate skin colour.

From this study, we can conclude that Campo de Hockey is playing a prominent role in increasing our understanding about early Iberian and European Megalithism especially due to the small number of megalithic necropolises for which reliable short-life sample radiocarbon dates are available in the Iberian Peninsula. The dimensions of the necropolis (53 burials), the good state of preservation of the human remains, and the diversity of grave goods found, have yielded valuable information about the community responsible for building the earliest megalithic tombs in southern Europe. This necropolis is exceptional due to the presence of up to four proto-megalithic graves and their chronology. The Bayesian model allowed us to determine the period during which the necropolis was active (approximately 300 years, in two phases). The four proto-megalithic graves belong to the so-called Phase I (4050/3960/3985–3900 cal BC). The presence of an even earlier proto-megalithic grave (dated to 4335–4060 cal BC) is even more remarkable. Phase II (3990–3755/3895–3620 cal BC) was characterised by the absence of proto-megalithic graves.

Regarding the age and sex distribution, a number of elements must be highlighted. All the human remains found inside proto-megalithic tombs are adult males. Subadult individuals were buried in the simplest features, and they constitute a large proportion of the total assemblage, which is indicative both of high infant mortality and of the fact that subadult individuals were regarded as deserving of funeral treatment (neonates burial patterns are unknown and therefore we can´t rule any hypothesis over their treatment in the community). The concentration of subadult graves (only this age group is documented in this area), to the north of the most monumental tomb in the necropolis (E11T14) also needs to be emphasised.

The maritime origin of Megalithism is heavily discussed. Some authors argue for a maritime expansion of megalithic concepts from their original locus in NW France, in relation to advanced seafaring technologies in the 5th millennium^12^. In this context, Campo de Hockey suggests the emergence of Megalithism in the Atlantic coast earlier than in the interior, in association with the operation of (maritime) distribution networks, which would be responsible for the arrival of exotic goods. Indeed, the arrival of these goods (sillimanite, amber, green stone, etc.) to the site supports the notion of navigation as an important activity, especially since some of these products (i.e., Sicilian amber), must have arrived to the Bay of Cádiz by sea.

## Supplementary Information

Supplementary Information.

## References

[1] Montelius O (1905). Orienten och Europa. Antiq Tidskr Sver.

[2] Childe VG (1950). Prehistoric Migrations in Europe.

[3] Bosch Gimpera, P. *Arqueología prerromana hispánica* (ed. Schulten, A.) 148–155 (Hispania, 1920).

[4] Bosch Gimpera P (1932). Etnología de la península Ibérica.

[5] Leisner, G. & Leisner, V. *Die Megalithgräber der Ibersichen Halbinsel: Der Süden* (Röm. Germ. Forsch. 17, 1943).

[6] Daniel G (1970). Megalithic answers. Antiquity.

[7] Blance B (1961). Early bronze age colonists in Iberia. Antiquity.

[8] Arribas A, Molina F, Fortea FJ, Jordá F (1984). Estado actual de la investigación del megalitismo en la península ibérica. Scripta Praehistorica. Francisco Jordá Oblata.

[9] Lucas Pellicer, R. El fenómeno megalítico: Estado actual de la investigación. In *Actas de la Mesa Redonda sobre Megalitismo Peninsular* (coord. Muñoz, G.) 11–20 (Asociación Española de Amigos de la Arqueología, 1986).

[10] López-Romero, E. Megalitismo y monumentalidad en la Prehistoria de la Península Ibérica. In *La Prehistoria en la Península Ibérica* (coord. López, P.) 439–536 (Itsmo-Akal, 2017).

[11] Renfrew C (1973). Before Civilization: The Radiocarbon Revolution and Prehistoric Europe.

[12] Schulz Paulsson B (2019). Radiocarbon dates and Bayesian modeling support maritime diffusion model for megaliths in Europe. Proc. Natl. Acad. Sci..

[13] Garrido Pena R, Rojo Guerra MA, Tejedor Rodríguez C, García Martínez De Lagrán I, Rojo MA, Garrido R, García I (2012). Capitulo IX. Las máscaras de la muerte: ritos funerarios en el Neolítico de la Península Ibérica. El Neolítico en la Peninsula Ibérica y su contexto europeo.

[14] Díaz-Zorita Bonilla M, Costa Caramé ME, García Sanjuán L, Gibaja JF, Carvalho AF, Chambon P (2012). Funerary practices from the Mesolithic to the Copper Age in Southern Spain. Funerary practices in the Iberian Peninsula from the Mesolithic to the Chalcolithic.

[15] Molina González, F., Cámara Serrano, J. A. & López Sáez, J. A. Andalucía. In *El Neolítico en la Peninsula Ibérica y su contexto europeo* (eds. Rojo, M. A., Garrido, R. & García, I.) 407–461 (Cátedra, 2012).

[16] Vijande E (2009). El poblado de Campo de Hockey (San Fernando, Cádiz): resultados preliminares y líneas de investigación futuras para el conocimiento de las formaciones sociales tribales en la Bahía de Cádiz (tránsito V-IV milenios a.n.e.). Revista Atlántica-Mediterránea de Prehistoria y Arqueología Social.

[17] Vijande E, Domínguez-Bella S, Cantillo JJ, Martín J, Barrena A (2015). Social inequalities in the Neolithic of South Europe: The grave goods of the Campo de Hockey necropolis (San Fernando, Cádiz, Spain). C.R. Palevol.

[18] Arteaga O, Schulz H, Roos AM (2008). Geoarqueología Dialéctica en la Bahía de Cádiz. Geoarqueología y proceso histórico en la Bahía de Cádiz, Revista Atlántica-Mediterránea de Prehistoria y Arqueología Social.

[19] Alonso C, Gracia FJ, Benavente J (2009). Evolución histórica de la línea de costa en el sector meridional de la Bahía de Cádiz. Revista Atlántica-Mediterránea de Prehistoria y Arqueología Social.

[20] Cantillo JJ, Vijande E, Cantillo JJ, Bernal D, Ramos J (2014). Análisis microespacial de la malacofauna marina en el asentamiento neolítico de Campo de Hockey (San Fernando, Cádiz). Nuevos datos sobre la función social del espacio. Molusco y púrpura en contextos arqueológicos atlántico-mediterráneos.

[21] Uzquiano P (2021). Mid-Holocene palaeoenvironmental record from the Atlantic Band of Cadiz (SW Spain) based on pollen and charcoal data. Quatern. Int..

[22] Van Klinken GJ (1999). Bone collagen quality indicators for palaeodietary and radiocarbon measurements. J. Archaeol. Sci..

[23] Reimer PJ (2020). The IntCal20 Northern Hemisphere radiocarbon age calibration curve (0–55 cal kBP). Radiocarbon.

[24] Heaton TJ (2020). Marine20—The marine radiocarbon age calibration curve (0–55000 cal BP). Radiocarbon.

[25] Fernandes, R., Larsen, T., Knipper, C., Feng, F. & Wang, Y. IsoMemo.com: A database of isotopic data for ecology, archaeology, and environmental sciences. https://isomemoapp.com/app/iso-memo-app. Accessed 5 May 2020 (2018).

[26] Cubas M (2019). Long-term dietary change in Atlantic and Mediterranean Iberia with the introduction of agriculture: A stable isotope perspective. Archaeol. Anthropol. Sci..

[27] Bronk Ramsey C (2009). Bayesian analysis of radiocarbon dates. Radiocarbon.

[28] Ward G, Wilson S (1978). Procedures for comparing and combining radiocarbon age determinations: A critique. Archaeometry.

[29] Ferembach D, Schwidetzsky I, Stloukal M (1979). Recommandations pour determiner l'age et le sexe sur le squelette. Bulletin el Mémoires de la Societé d´Anthropologie de Paris.

[30] Buikstra, J. & Ubelaker, D. *Standards for Data Collection from Human Skeletal Remmains: Proceedings of a seminar at the Museum of Natural History* (Arkansas Archaeological Survey, 1994).

[31] Alemán I, Botella MC, Ruiz L (1997). Determinación del sexo en el esqueleto postcranial. Estudio de una población mediterránea actual. Arch. Esp. Morfol..

[32] Meindl RS, Lovejoy CO (1985). Ectocranial suture closure: A revised method for the determination of skeletal age at death based on the lateral-anterior sutures. Am. J. Phys. Anthropol..

[33] Brooks S, Suchey JM (1990). Skeletal age determination based on the os pubis: a comparison of the Acsádi-Nemeskéri and Suchey-Brooks methods. Hum. Evol..

[34] Lovejoy CO, Meindl RS, Mensforth R, Barton TJ (1985). Chronological metamorphosis of the auricular surface of the ilium: A new method for the determination of adult skeletal age at death. Am. J. Phys. Anthropol..

[35] Schutkowski H (1993). Sex determination of infant and juvenile skeletons: I. Morphognostic features. Am. J. Phys. Anthropol..

[36] Loth SR, Henneberg M (2001). Sexually dimorphic mandibular morphology in the first few years of life. Am. J. Phys. Anthropol..

[37] Alqahtani SJ, Hector MP, Liversidge HM (2010). Brief communication: the London atlas of human tooth development and eruption. Am. J. Phys. Anthropol..

[38] Schaefer M, Black S, Scheuer L (2009). Juvenile Osteology.

[39] Olalde I (2019). The Genomic History of the Iberian Peninsula over the Past 8000 Years. Science.

[40] Skoglund P, Storå J, Götherström A, Jakobsson M (2013). Accurate sex identification of ancient human remains using DNA shotgun sequencing. J. Archaeol. Sci..

[41] Kennett DJ (2017). Archaeogenomic evidence reveals prehistoric matrilineal dynasty. Nat. Commun..

[42] Monroy Kuhn JM, Jakobsson M, Günther T (2018). Estimating genetic kin relationships in prehistoric populations. PLoS ONE.

[43] Patterson N, Price AL, Reich D (2006). Population structure and eigenanalysis. PLoS Genet..

[44] Lazaridis I (2014). Ancient human genomes suggest three ancestral populations for present-day europeans. Nature.

[45] Lazaridis I (2016). Genomic insights into the origin of farming in the ancient near east. Nature.

[46] Patterson N (2012). Ancient admixture in human history. Genetics.

[47] Haak W (2015). Massive migration from the steppe was a source for Indo-European languages in Europe. Nature.

[48] Ringbauer, H., Novembre, J. & Steinrücken, M. Detecting runs of homozygosity from low-coverage ancient DNA. *BioRxiv* **2020.05.31.126912**. 10.1101/2020.05.31.126912 (2020).

[49] Chaitanya L (2019). Genetics the HIrisPlex-S system for eye, hair and skin colour prediction from DNA : Introduction and forensic developmental validation. Forensic Sci. Int..

[50] Domínguez-Bella S, Ramos-Muñoz J, Pérez M, Ramos J (2008). Productos arqueológicos exóticos en los contextos de los yacimientos prehistóricos de la Banda Atlántica de Cádiz. Inferencias de su documentación. La ocupación prehistórica de la campiña litoral y banda atlántica de Cádiz. Aproximación al estudio de las sociedades cazadoras-recolectoras y tribales-comunitarias y clasistas iniciales.

[51] Guilaine J (2002). Matériaux, productions, circulations du Néolithique à l’Âge du Bronze.

[52] Domínguez-Bella, S., Morata, D., De La Rosa, J. & Ramos, J. Neolithic trade routes in SW Iberian Peninsula? Variscite green beads from some Neolithic sites in the Cádiz province (SW Spain): raw materials and provenance areas. In *Proceedings of 32 International Symposium on Archaeometry. México D.F. 2000* (eds. Bautista, P. & Barba, L.) Electronic Book (Universidad Nacional Autónoma de México, 2002).

[53] Domínguez-Bella S (2004). Variscite, a prestige mineral in the Neolithic-Aeneolithic Europe. Raw material sources and possible distribution routes, Slovak Geological Magazine.

[54] Cassen S, Müller J, Furholt M (2011). Measuring distinction inside the architectures of the Carnac region: From sign to material. Megaliths and Identities. Frühe Monumentalität und soziale Differenzierung.

[55] Cassen S, Pétrequin P (2012). Dêpots bretons, tumulus carnacéens et circulations à longue distance. Grandes haches alpines du Néolithique européen. Ve et IVe millénaires av. J.-C..

[56] Querré G, Domínguez-Bella S, Cassen S, Querré G, March-Hand G (2012). La variscite ibérique. Exploitation, diffusion au cours du Néolithique. Roches et Sociétés de la Préhistoire. Entre massifscristallins et bassins sédimentaires. Le nord-ouest de la France dansson contexte européen.

[57] Beck CW (1986). Spectroscopic investigations of amber. Appl. Spectrosc. Rev..

[58] Devièse T (2017). A multi-analytical approach using FTIR, GC/MS and Py-GC/MS revealed early evidence of embalming practices in Roman catacombs. Microchem. J..

[59] Murillo-Barroso M (2018). Amber in prehistoric Iberia: New data and a review. PLoS ONE.

[60] Odriozola CP (2019). Amber, beads and social interaction in the Late Prehistory of the Iberian Peninsula: an update. Archaeol. Anthropol. Sci..

[61] Emslie SD (2019). Mercury in archaeological human bone: biogenic or diagenetic?. J. Archaeol. Sci..

[62] Yáñez C (2002). El mon funerary al final del V millenni a Andorra: la Tomba de Segudet (Ordino). Cypsela.

[63] MorellRovira B (2018). Tracing the chronology of neolithic pit and stone box burials in North-eastern Iberia. J. Archaeol. Sci. Rep..

[64] Alonso Matthias F, Bello Diéguez JM, Rodríguez A (1997). Cronología y periodización del fenómeno megalítico en Galicia a la luz de las dataciones por Carbono 14. O Neolítico Atlántico e as orixes do megalitismo.

[65] Fábregas Valcarce, R. & Vilaseca Vázquez, X.I. En torno al megalitismo gallego. In *Arte parietal megalítico en el noroeste peninsular: conocimiento y conservación* (coords. Carrera, F. & Fábregas, R.) 11–36 (Tórculo, 2006).

[66] SchulzPaulsson B (2017). Time and Stone: The Emergence and Development of Megaliths and Megalithic Societies in Europe.

[67] Mujika JA, Edeso JM (2011). Los primeros agricultores y ganaderos en Gipuzkoa del Neolítico a la Edad del Hierro.

[68] Diniz M, DeSenna-Martínez J-C, Diniz M, Faustino A (2018). The origins of Megalitism in Western Iberia: Resilient signs of a symbolic revolution?. The Gibraltar aos Pirenéus, Megalitismo, Vida e Morte na Fachada Atlântica Peninsular.

[69] Mataloto, R., Andrade, M. & Pereira, A. O Megalitismo das pequenas antas: novos dados para um velho problema. *A Rui Boaventura. Homenagem à sua Memória, Estudos Arqueológicos de Oeiras* **23**, 33–156 (Câmara Municipal, 2016–2017).

[70] García Sanjuán L (2020). Builders of Megaliths: Society, monumentality and environment in 4th millennium cal BC Antequera. J. Archaeol. Sci. Rep..

[71] Ramos J, Giles F (1996). El dolmen de Alberite (Villamartín) Aportaciones a las formas económicas y sociales de las comunidades neolíticas en el noroeste de Cádiz.

[72] Gibaja J (2017). The Chronology of the Neolithic Necropolis Bòbila Madurell-Can Gambús in the Northeast Iberian Peninsula: Dating the Pit Burials Cultural Horizon and Long-Range Raw Material Exchange Networks. Radiocarbon.

[73] Roig J (2010). The necropolis of Can Gambus-1 (Sabadell, Barcelona). New data on the funerary practices during the Middle Neolithic in the northeast of the Iberian Peninsula. Trab. Prehist..

[74] García Gazólaz, J. & Sesma, J. Los enterramientos neoliticos del yacimiento de Paternanbidea (Ibero). In *La tierra te sea leve. Arqueología de la muerte en Navarra. Catálogo de la exposición* 59–65 (Gobierno de Navarra, 2007).

[75] García Gazólaz, J. & Sesma, J. Enterramientos en el poblado neolítico de Los Cascajos (Los Arcos, Navarra). In *La tierra te sea leve. Arqueología de la muerte en Navarra. Catálogo de la exposición* 52–58 (Gobierno de Navarra, 2007).

[76] Rojo M, Kunst M, Garrido R, García I, Morán G (2005). Un desafío para la eternidad: tumbas monumentales del Valle de Ambrona.

[77] Rojo Guerra M, Garrido R, Martínez de Lagrán I (2010). Tombs for the dead, monuments to eternity: the deliberate destruction of megalithic graves by fire in the interior highlands of Iberia (Soria province, Spain). Oxford J. Archaeol..

[78] Bueno Ramírez P, Barroso Bermejo R, de Balbín Behrmann R, Laporte L, Scarre C (2016). Between east and west: Megaliths in the centre of the Iberian Peninsula. The Megalithic Architectures of Europe.

[79] Soares J, Da Silva CT, Gonçalves VS (2000). Protomegalitismo no sul de Portugal: inauguraçao das paisagens megalíticas. Muitas antas, pouca gente? Actas do I Colóquio Internacional sobre Megalitismo.

[80] Leandri F, Gilabert C, Demouche F, Moinat P, Chambon P (2007). Les chambres funéraires du Ve millénaire av. J.-C.: le cas de la Corse. Les cistes de Cham blandes et la place des coffres dans les pratiques funéraires du Néolithique moyen occidental.

[81] Domínguez Bella, S., Álvarez Rodríguez, M. A. & Ramos Muñoz, J. Estudio analítico de las cuentas de collar de ámbar del dolmen de Alberite (Villamartín, Cádiz). Naturaleza química y mineralógica e implicaciones sobre su origen. In *3º Congreso Nacional de Arqueometría (Sevilla)*, 621–630. http://hdl.handle.net/11441/67105 (Universidad de Sevilla, 1999).

[82] Hoskin M (2020). Tumbas, templos y sus orientaciones: una nueva perspectiva sobre la Prehistoria del Mediterráneo.

[83] Sánchez-Barba L (2019). possible interpersonal violence in the Neolithic necropolis of Campo de Hockey (San Fernando, Cádiz, Spain). Int. J. Paleopathol..

[84] Lipson M (2017). Parallel ancient genomic transects reveal complex population history of early European farmers. Nature.

[85] Martiniano R (2017). The population genomics of archaeological transition in West Iberia: Investigation of ancient substructure using imputation and haplotype-based methods. PLoS Genet..

[86] Valdiosera C (2018). Four millennia of Iberian biomolecular prehistory illustrate the impact of prehistoric migrations at the far end of Eurasia. Proc. Natl. Acad. Sci. USA.

[87] Fu Q (2016). The genetic history of ice age Europe. Nature.

